# The DizzyQuest: relation between self-reported hearing loss, tinnitus and objective hearing thresholds in patients with Meniere’s disease

**DOI:** 10.1007/s00415-021-10909-8

**Published:** 2021-12-11

**Authors:** E. C. Martin, R. Verkaik, J. J. A. Stultiens, M. R. van de Berg, A. M. L. Janssen, C. Leue, P. Delespaul, F. Peeters, J. Widdershoven, A. Erdkamp, S. C. F. van de Weijer, H. Blom, A. Zwergal, E. Grill, N. Guinand, A. Perez-Fornos, D. Tse, R. van de Berg

**Affiliations:** 1 Division of Balance Disorders, Department of Otorhinolaryngology and Head and Neck Surgery, Maastricht University Medical Center, Maastricht, The Netherlands; 2 Department of Methodology and Statistics, Maastricht University Medical Center, Maastricht, The Netherlands; 3 Department of Psychiatry and Neuropsychology, School for Mental Health and Neuroscience, Maastricht University Medical Center, Maastricht, The Netherlands; 4 Department of Clinical Psychological Science, Faculty of Psychology and Neuroscience, Maastricht University, Maastricht, The Netherlands; 5 Department of Psychiatry and Psychology, Maastricht University Medical Center, Maastricht, The Netherlands; 6 Department of Otolaryngology—Head and Neck Surgery, HagaZiekenhuis, The Hague, The Netherlands; 7 Department of Neurology, Ludwig-Maximilians-University of Munich, Munich, Germany; 8 Department of Medical Informatics, Ludwig-Maximilians-University of Munich, Munich, Germany; 9 Service of Otorhinolaryngology—Head and Neck Surgery, Department of Clinical Neurosciences, Geneva University Hospitals, Geneva, Switzerland; 10 Department of Otolaryngology—Head and Neck Surgery, University of Ottawa, The Ottawa Hospital, Civic Campus, Ottawa, Canada; 11 Faculty of Physics, Tomsk State Research University, Tomsk, Russia

**Keywords:** DizzyQuest, Meniere’s disease, Tinnitus, Hearing loss, Audiometry, Experience sampling

## Abstract

**Background:**

Combining a mobile application-based vestibular diary called the DizzyQuest and an iPad-based hearing test enables evaluation of the relationship between experienced neuro-otological symptoms and hearing thresholds in daily life setting. The aim was to investigate the relationship between self-reported hearing symptoms and hearing thresholds in patients with Meniere’s disease (MD), using the DizzyQuest and the iPad-based hearing test simultaneously.

**Methods:**

The DizzyQuest was administered for 3 weeks in 21 patients. Using the experience-sampling-method (ESM), it assessed hearing loss and tinnitus severity for both ears separately. Each day after the DizzyQuest, an iPad-based hearing test was used to measure hearing thresholds. A mixed model regression analysis was performed to investigate relationships between hearing thresholds and self-reported hearing loss and tinnitus severity.

**Results:**

Fifteen patients were included. Overall, pure-tone averages (PTAs) were not correlated with self-reported hearing loss severity and tinnitus. Individual differences in PTA results between both ears did not significantly influence the difference in self-reported hearing loss severity between both ears. Self-reported hearing loss and tinnitus scores were significantly higher in ears that corresponded with audiometric criteria of MD (*p* < 0.001). Self-reported tinnitus severity significantly increased with self-reported hearing loss severity in affected (*p* = 0.011) and unaffected ears (*p* < 0.001).

**Conclusion:**

Combining the DizzyQuest and iPad-based hearing test, facilitated assessment of self-reported hearing loss and tinnitus severity and their relationship with hearing thresholds, in a daily life setting. This study illustrated the importance of investigating neuro-otological symptoms at an individual level, using multiple measurements. ESM strategies like the DizzyQuest should therefore be considered in neuro-otological research.

**Supplementary Information:**

The online version contains supplementary material available at 10.1007/s00415-021-10909-8.

## Introduction

Meniere’s disease (MD) is an inner ear disease that results in attacks of vertigo, combined with fluctuating ear symptoms like tinnitus, aural fullness and sensorineural hearing loss. The prevalence is estimated around 34–190 in 100,000 people [1, 2] and it can have a strong socio-economic impact, depending on the severity of the disease. Especially in the early stage of the disease, hearing levels can fluctuate [2]. Often in a later stage, permanent sensorineural hearing loss occurs. Fluctuating hearing loss is reported by patients, but is often not well documented due to logistic reasons: reliable audiometry cannot be performed at any time or place. This creates a diagnostic challenge, since audiometrically documented sensorineural hearing loss is a diagnostic criterium for MD [2]. To overcome this challenge, other options should be considered. At this moment, many unvalidated mobile applications (apps) are available, but recently a mobile iPad-based hearing test was validated [3–5]. This facilitates reliable measurements of hearing function at any time, in the patients’ own environment.

Furthermore, a new app-based vestibular diary was recently introduced by the Dizzynet network [6, 7]: the DizzyQuest. It comprises standardized questionnaires which are able to capture vestibular symptoms and their daily life psychosocial context. It includes, among others, disease-specific and generic questions regarding dizziness, hearing, tinnitus and their impact on quality of life [8]. It is based on the experience sampling method (ESM), which implies that multiple measurements during daily life in the patient’s own environment are performed. This results in a high accuracy (little recall bias) and a high ecological validity (data reflect the way symptoms appear in real-life settings), compared to many currently used questionnaires for vestibular disorders [9]. By combining the mobile hearing test with the DizzyQuest, the relationship between hearing thresholds and self-reported symptom severity might be explored.

The objective of this study was to investigate the relationship between self-reported hearing symptoms and hearing thresholds in MD patients, using the DizzyQuest and the iPad-based hearing test simultaneously. It was hypothesized that a significant relationship would exist between individual hearing threshold differences and corresponding differences in self-reported hearing symptoms. Overall, a weaker correlation was expected between hearing thresholds and self-reported hearing symptoms due to interpersonal differences in symptom experience and coping mechanisms [10–12].

## Materials and methods

### Patients

MD patients with audiovestibular symptoms including fluctuating sensorineural hearing loss, were recruited by the Department of Otorhinolaryngology and Head & Neck Surgery of Maastricht University Medical Center+ (Maastricht, The Netherlands) and Haga hospital (Den Haag, The Netherlands). Inclusion criteria comprised: aged 18 years or older, proficient in Dutch language and basic understanding of English texts, diagnosed with MD by an otorhinolaryngologist according to the Barany Society criteria [2] and in possession of a smartphone or tablet (operating system Android OS 4.0 or iOS 8.0, or newer) with an internet connection. Patients were excluded if they did not feel comfortable answering questions from the DizzyQuest (e.g., questions about psychosocial context).

### DizzyQuest

The DizzyQuest is an app-based diary for vestibular patients (www.dizzyquest.com). It runs from the UM ESM (v2.04) application. This application is an experimental version of the PsyMate™ app (www.psymate.eu) and can be downloaded on a smartphone or tablet. It acts as a platform for medical research that enables the administration of multiple questionnaires and sampling schemes. The DizzyQuest consists of four different questionnaires, of which only the evening questionnaire was used for this study. The evening questionnaire reflects on the past day using questions about vestibular symptoms and their psychosocial context (see Supplementary Material 1). A seven-point Likert scale is used to grade the symptoms from 1 (not at all) to 7 (very much). The other DizzyQuest questionnaires (day-, morning- and attack-questionnaires) were not suited for this study due their nature, timing and frequency at which they were administered [9].

### Hearing test

#### Equipment

SHOEBOX Audiometry’s (SHOEBOX Audiometry Ltd., Ottawa, Canada) automated audiometer on iPad (Apple Inc., Cupertino, CA, USA) was used to conduct air conduction audiometry. The following settings were used: noise alert mode off, masked air testing on, no Maximum Permissible Ambient Noise Level protocol, minimum volume 0 dB, and maximum volume 90 dB. RadioEar (RadioEar, Middelfart, Denmark) DD450 headphones were used for testing air conduction thresholds [4]. All devices were calibrated according to ANSI s3.6/EN 60,645–1 standards using proprietary Calibration Software and Hardware. Calibration was valid for a period of 1 year and after that, they were recalibrated [3].

#### Audiometric testing paradigm

Air conduction hearing thresholds were measured for tonal stimuli at 250, 500, 1000, 2000, 3000, 4000 and 8000 Hz. The right ear was always tested first. A play task format was used for this hearing test [3, 13]. Audiograms were saved locally and automatically uploaded to an online personal account when the device connected to Wi-Fi*.*

### Dizziness handicap inventory and hospital anxiety and depression scale questionnaires

Patients were instructed to fill in the Dizziness Handicap Inventory (DHI) [14] and Hospital Anxiety and Depression Scale (HADS) [15] questionnaires at baseline. The DHI consists of 25 questions focussing on the self-perceived handicap in daily life, due to dizziness. It is subdivided in 3 domains in which a specific number of points can be obtained (28 for the physical domain, 36 for the emotional domain and 36 for the functional domain). For this study, domain scores were summed, with a maximum total score of 100 points. The total score indicates the self-perceived handicap due to dizziness: 0–30 mild handicap; 31–60 moderate handicap; 61–100 severe handicap.

The HADS is a questionnaire to screen for states of anxiety and depression in outpatients. It consists of 14 multiple choice questions of which 7 focus on anxiety and 7 on depression. Depending on the answer, 0, 1, 2 or 3 points can be scored per question. A higher score indicates a greater risk of anxiety or depression. Total scores are summed for anxiety and depression separately. The following cut-off scores were used: 0–7 for none, 8–10 for possible and > 11 for probable anxiety or depression [15].

### Study design

Patients were invited to participate in a 3-week trial. At baseline, the DHI and HADS questionnaires were completed. Then, patients were asked to complete the DizzyQuest and perform the iPad-based hearing test each day consecutively during the trial period. Before the trial, patients were contacted by two of the authors (AE, SvdW) and received an instructional video on how to use the DizzyQuest. Next to this, other authors (RV, EM, JS, MvdB) instructed them on how to use the iPad-based hearing test, and a written instruction for future reference was provided. The necessity of a quiet testing environment was emphasized.

Each day during the 3-week trial, the evening questionnaire became available in the DizzyQuest at 7:30 PM. Patients were alerted by a notification on their phone at 8 PM. The evening questionnaire remained available until 4:30 AM the next morning. Immediately after completing the evening questionnaire, the iPad-based hearing test had to be performed. This order was chosen to prevent anchoring bias (audiometric results were shown directly after completion of the test). In case a patient did not complete the hearing test, this was considered as missing data since average PTAs could not be calculated for that given day.

#### Selected questions from the DizzyQuest

Five questions from the DizzyQuest evening questionnaire were selected: (1) Today I suffered from hearing loss: left; (2) Today I suffered from hearing loss: right; (3) Today I suffered from tinnitus: left; (4) Today I suffered from tinnitus: right; (5) Today I suffered from sensitivity to sounds”. This selection was made in consensus between two authors (RV, RvdB) and was based on their theme: aural symptoms. The first four questions regarding hearing loss and tinnitus were included as main outcome parameters. The fifth question “I suffered from sensitivity to sound” was included to analyse a possible confounding effect of hyperacusis, since severe self-reported handicap of tinnitus is closely associated with severity of hyperacusis [16].

### Data preparation

#### Hearing test results

In case it was very clear that the left and right side of the headphones were accidentally switched by the patient (since hearing thresholds of both ears matched contralateral thresholds measured during other days), hearing thresholds were corrected for their side. Suspected hearing test artefacts (e.g., thresholds resembling a deaf ear without any report in the DizzyQuest, probably due to an audio problem of the iPad) were excluded from analysis.

#### Affected versus non-affected ear

It was checked for each patient whether they audiometrically fulfilled the diagnostic criteria for MD during the study period. For this, the audiometric criteria for MD were applied to the obtained iPad-based hearing test results of each ear [2]. For each patient, this could result in one side affected, both sides affected, or both sides unaffected. “Unaffected” only implied that during the study period the obtained hearing tests results did not comply with the diagnostic criteria for MD. However, all patients had already fulfilled the criteria previously, in order to be included in this study.

### Data analysis

Patients were selected for analysis if they had at least 10 “valid testing days”. A valid testing day was considered when the hearing test was completed to such extent that the main outcome parameters could be analysed. This involved obtainment of all hearing thresholds on the octave frequencies of 500–4000 Hz to calculate the Pure Tone Average (PTA) on both ears.

Data were analysed with IBM SPSS Statistics 25 [17]. For every day, answers to four questions (regarding hearing loss and tinnitus) of the DizzyQuest evening questionnaire were correlated to the corresponding PTA scores of that day.

To visually explore the different relationships, scatterplots per patient were made. Since every patient was tested multiple times, the assumption of independence was violated. Therefore, mixed effects regression analysis was used to analyse the data. The most appropriate model was selected using likelihood ratio testing. To correct (adjust) for a possible confounding effect of hyperacusis (“sensitivity to sounds”, Likert score) and time (number of testing days) separate analyses were performed including these variables in addition to the variable of interest. A significance level of 0.05 was applied.

Finally, hearing tests obtained with the iPad-based at home, were compared to previously obtained hearing tests from an audiological center which were part of routine clinical care. For each patient, hearing thresholds per octave frequency (500–1000–2000–4000 Hz) of a hearing test obtained at an audiological center, were compared to the average hearing thresholds per octave frequency of the iPad-based hearing test. The time difference between the hearing test in the audiological center and the first day of the iPad-bed hearing test, was calculated in days. Data analysis was performed by calculating the Correlation Coefficient (ICC) using a single-measure, two-way mixed-effects model (absolute agreement) [18]. Mean ICC along with 95% confidence intervals (CI) were reported. ICC values less than 0.5, between 0.5 and 0.75, between 0.75 and 0.9, and greater than 0.9 were considered as poor, moderate, good and excellent reliability, respectively [19].

### Ethical considerations

Approval for this study was given by the research ethics board of Maastricht University Medical Center (MUMC+) (protocol: METC 2018-0809). Informed consent was obtained from all patients through the DizzyQuest application.

## Results

### Patient characteristics and response rate

Twenty-one patients were enrolled in this study. Three patients were lost to follow-up (personal reasons, symptom severity) and three patients were excluded since they completed less than 10 valid testing days. Therefore, data obtained from 15 patients was suited for analysis. Tables 1 and 2 show the patient characteristics of these 15 patients. It involved seven men and eight women, with an average age of 65 years old (range 57–80 years). These patients provided on average 18 valid testing days (range 10–21 days) and the mean duration of MD symptoms was 138 months (range 15–348 months). Twelve out of 15 patients reported a moderate self-perceived handicap, while 2 reported a severe handicap. In 11 patients, anxiety and/or depression were considered to be present (HADS subdomain score > 11, Table 2).

**Table 1:** Patient characteristics

Patient	Sex	Age (years)	Duration of MD (months)^a^	Affected ear(s) (MD)	Affected ear(s) study	Number of valid testing days
1	Female	61	54	Left	Left	19
2	Male	73	204	Both	Both	15
3	Female	57	168	Both	Both	19
4	Female	63	120	Both	None	21
5	Male	79	96	Both	Both	21
6	Female	69	120	Right	Right	19
7	Male	75	96	Right	Right	10
8	Female	61	38	Left	Left	20
9	Male	63	120	Both	Right	20
10	Female	61	120	Both	None	20
11	Male	71	288	Both	Left	20
12	Male	66	114	Right	Right	13
13	Male	66	15	Left	Left	17
14	Female	68	132	Both	Left	14
15	Female	41	348	Left	Left	18

^a^Approximate duration of MD in months. “Affected ear(s) (MD)” involves the ears that were classified as MD in the past. This does not imply that they audiometrically fulfilled the MD criteria during the study period. Therefore, ears that did fulfil the MD criteria during the study period, are listed in the column “Affected ear(s) study”

**Table 2:** Baseline individual scores of the Dizziness Handicap Inventory (DHI) and the Hospital Anxiety and Depression scale (HADS)

Patient	DHI	HADS anxiety	HADS depression
1	43	12	10
2	52	11	12
3	53	14	9
4	48	12	9
5	46	10	7
6	46	11	14
7	47	11	6
8	43	10	6
9	52	10	10
10	50	9	11
11	41	12	10
12	70	15	12
13	65	13	9
14	30	13	10
15	42	10	9

### Relationship between PTA and self-reported hearing loss severity

**Fig. 1:**
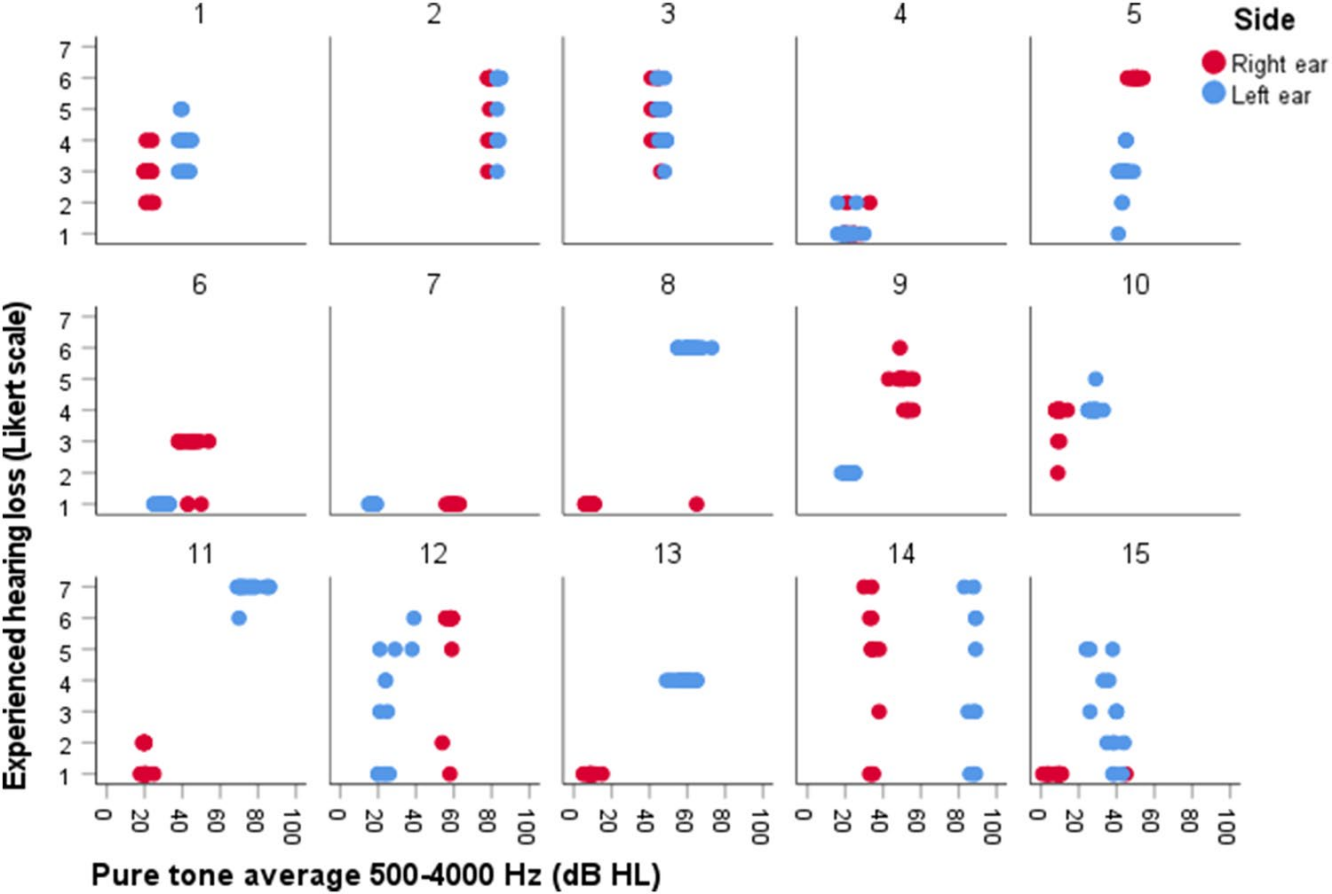
Relationship between objectively measured hearing thresholds (PTA 500–4000 Hz) with an iPad-based hearing test at home, and self-reported hearing loss severity using the DizzyQuest, in 15 MD patients (30 ears) during 3 weeks. A Likert scale was used to assess self-reported hearing loss severity, varying from 1 (not at all) to 7 (very much)

Figure 1 presents the relationship between PTA 500–4000 Hz (dB HL) measured with the iPad-based hearing test, and self-reported hearing loss severity measured with the DizzyQuest, in 15 MD patients (30 ears). Regarding PTA, it can be observed that on group level, PTAs between patients varied (e.g., patient 2 versus patient 4). On an individual level, PTAs of left and right ears could be close to each other (e.g., patients 2, 3, 4, 5) or differ substantially (e.g., patient 7, 8, 11, 13, 14). However, on the level of a single ear, most ears did not show much variability in PTA during the study period. Regarding self-reported hearing loss severity, this could vary between patients with almost the same PTAs (e.g., patient 9 versus patient 12) and between ears of the same patient (e.g., patient 7). Therefore, between and within patients, the same PTA did not necessarily lead to the same self-reported hearing loss severity (e.g., comparing reported hearing loss in patient 8 versus patient 13, both ears). Accordingly, mixed effects regression analysis revealed that PTA could not significantly predict self-reported hearing loss (*B* = 0.023, *t* (8.823) = 1.489, *p* = 0.171). Results were similar after adjusting for hyperacusis and number of testing days. However, self-reported hearing loss was significantly lower in the non-affected ears, compared to the affected ears: on average 2.4 points on the Likert scale (*B* = − 2.385, *t* (27.448) = − 4.713, *p* < 0.001). This effect was also comparable after adjusting for hyperacusis and number of testing days. When comparing both ears of individual patients, the PTA difference between both ears did not significantly influence the difference in self-reported hearing loss severity (*B* = 0.026, *t* (12.023) = 1.418, *p* = 0.182).

### Relationship between PTA and self-reported tinnitus severity

**Fig. 2:**
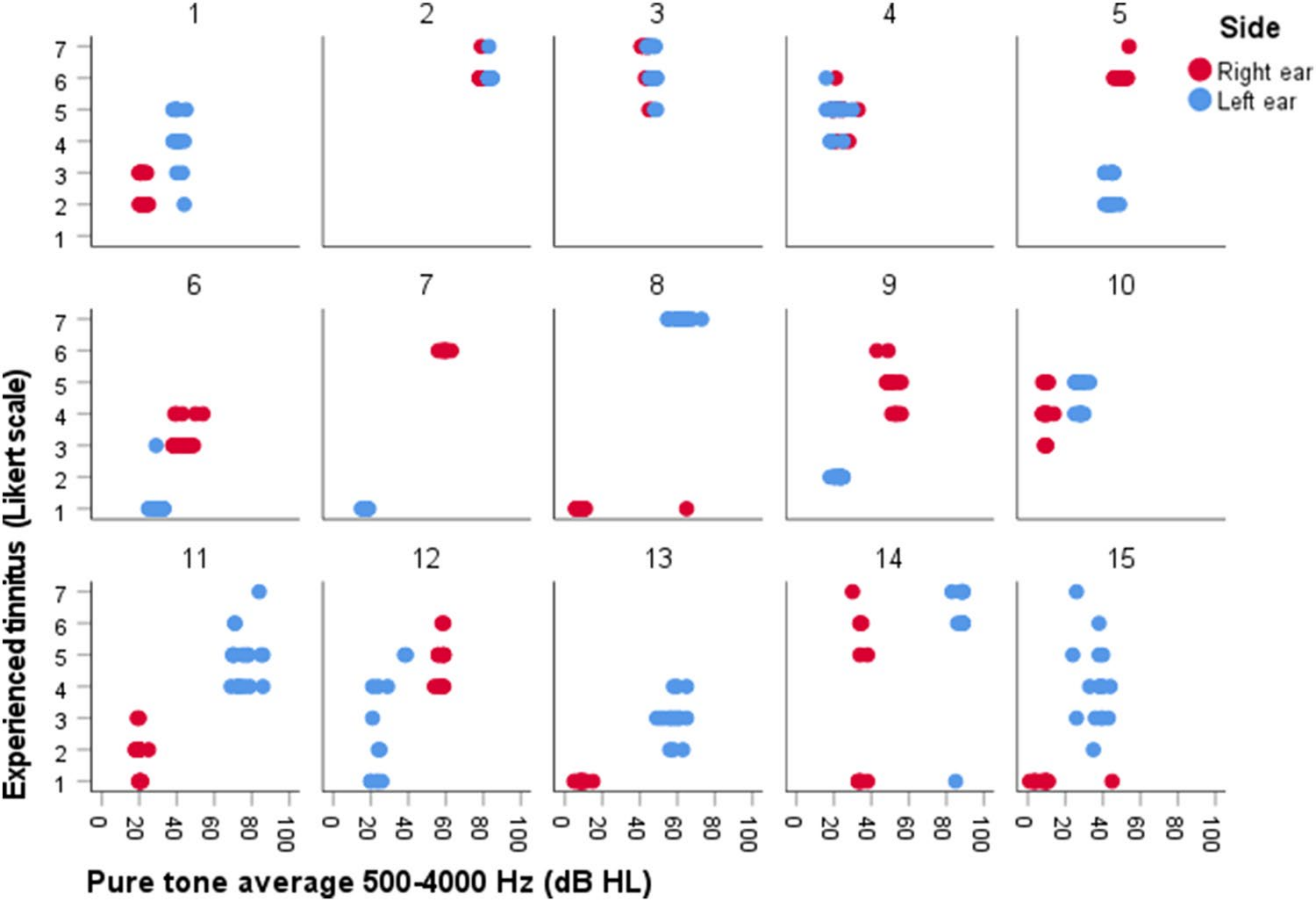
Relationship between objectively measured hearing thresholds (PTA 500–4000 Hz) with an iPad-based hearing test at home, and self-reported tinnitus severity using the DizzyQuest, in 15 MD patients (30 ears) during 3 weeks. A Likert scale was used to assess self-reported tinnitus severity, varying from 1 (not at all) to 7 (very much)

Figure 2 presents the relationship between PTA 500–4000 Hz (dB HL) measured with the iPad-based hearing test, and self-reported tinnitus severity measured with the DizzyQuest, in 15 MD patients (30 ears). Self-reported tinnitus severity could vary between patients (e.g., patient 8 versus patient 13), between ears of the same patient (e.g., patient 7, left versus right ear) and within the same ear (e.g., patient 15, left ear). The same PTA did not necessarily lead to the same self-reported tinnitus severity in different patients. Mixed effects regression analysis showed that PTA was not significantly related to the amount of self-reported tinnitus severity (*B* = 0.020, *t* (9.540) = 1.524, *p* = 0.160). Results were similar after adjusting for hyperacusis and number of testing days. In contrast, non-affected ears showed a significantly lower Likert score (on average 2.8 points) than affected ears (*B* = − 2.760, *t* (19.343) = − 6.335, *p* < 0.001). This effect was also comparable after adjusting for hyperacusis and number of testing days. No significant relationship was found between PTA difference and the difference in self-reported tinnitus severity between ears of the same patient (*B* = 0.030, *t* (9.707) = 1.881, *p* = 0.090).

### Relationship between self-reported hearing loss and tinnitus severity

**Fig. 3:**
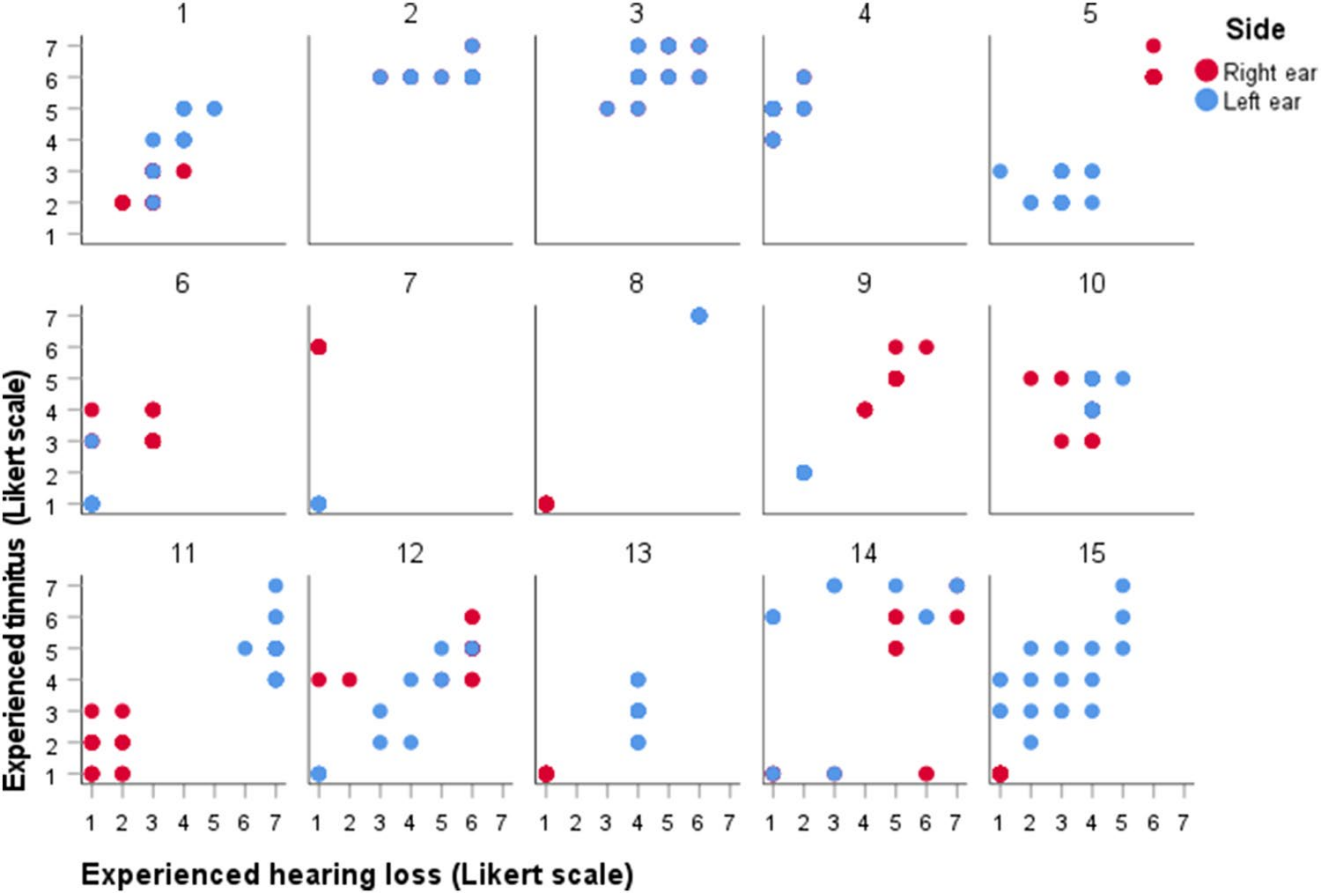
Relationship between self-reported hearing loss and tinnitus severity using the DizzyQuest, in 15 MD patients (30 ears) during 3 weeks. A Likert scale was used to assess self-reported hearing loss and tinnitus severity, varying from 1 (not at all) to 7 (very much)

Figure 3 presents the relationship between self-reported hearing loss and tinnitus severity measured with the DizzyQuest, in 15 MD patients (30 ears). In most patients, it can be observed that self-reported tinnitus severity seemed to increase with increasing severity of self-reported hearing loss (e.g., patients 1, 5, 8, 9, 11, 12, 15, etc.). Mixed effects regression analysis also showed a significant relationship between these two self-reported symptoms: self-reported tinnitus severity increased with self-reported hearing loss severity, although this was different for affected and unaffected ears (*B* = 0.270, *t* (13.033) = 2.963, *p* = 0.011 and *B* = 0.642, *t* (20,114) = 6.356, *p* < 0.001, respectively). Results were similar after adjusting for hyperacusis and number of testing days in affected and unaffected ears.

### Comparison of iPad-based hearing test results with hearing tests obtained at an audiological center

The time difference between the test at an audiological center and the start of the iPad-hearing test ranged from 5 days to 56 months (SD 558.8 days). The average hearing thresholds obtained with the iPad-based hearing test at home, showed a good correlation for both the right (ICC = 0.884, 95% CI [0.773, 0.937]) and left ears (ICC = 0.875, 95% CI [0.736, 0.935]) with the hearing thresholds obtained at audiological centers.

## Discussion

The aim of this study was to investigate the relationship between self-reported hearing symptoms and hearing thresholds in patients with Meniere’s disease (MD), using the DizzyQuest and the iPad-based hearing test simultaneously. This enabled the opportunity to perform multiple measurements in the same individuals, in daily life setting. It was shown that overall, PTAs were not correlated with self-reported severity of hearing loss and tinnitus. Furthermore, no significant relationship was found between individual PTA difference and corresponding difference in self-reported hearing loss or tinnitus severity between the two ears of the same subjects. However, self-reported hearing loss and tinnitus severity were on average reported to be higher in ears that (during this study period) corresponded with the audiometric criteria of MD, than ears that did not comply with the criteria. Additionally, self-reported tinnitus severity and self-reported hearing loss were significantly related: self-reported tinnitus severity increased with self-reported hearing loss severity. This study illustrates one of the positive aspects of using the ESM method: the same individuals are measured multiple times. Multiple measurements facilitate more reliable assessment of symptom severity on an individual level than when using only a single momentary assessment [20].

It was demonstrated that (relatively) objective measurements like PTAs do not correlate well with self-reported hearing loss or tinnitus, but that self-reported items between themselves, can correlate to a certain extent. The discrepancy between the (relatively) objective hearing tests and self-reported hearing loss is congruent with previous literature [21]. However regarding tinnitus, literature provides conflicting evidence regarding the correlation between hearing loss and self-reported tinnitus severity [22, 23]. Nevertheless, in case of a significant correlation, this was found to be weak [24]. The discrepancies between (relatively) objective tests and self-reported symptoms might show that both techniques measure different aspects and can be complementary to each other. The discrepancies are also likely attributed to the fact that groups are often heterogenous in baseline characteristics, including emotional and environmental factors that might influence the experienced burden of disease [25], relatively independent of objective findings. These factors might also explain the significant relationship between self-reported hearing loss and tinnitus severity found in this study. Surprisingly, hyperacusis did not significantly influence the self-reported hearing loss and tinnitus severity. This is in conflict with the current literature [16]. Although multiple other psychosocial factors were also measured by the DizzyQuest, it was beyond the scope of this article to investigate these types of relations.

Furthermore, no significant effect was found when comparing individual differences in hearing threshold with corresponding differences in experienced hearing loss or tinnitus between both ears of the same patient. However, this does not imply that no effect can be present.

Main challenge of this study was the fact that PTAs within the same ears did not show much variability during the study period. This was against expectations, since patients indicated to experience fluctuations in hearing loss at time of study inclusion. This finding demonstrates one of the strengths of prospective repeated measurements: recall bias is lowered. After all, it would be unlikely that patients suddenly stopped having fluctuations in hearing loss at time of inclusion. Most likely due to recall bias, episodes of fluctuations in hearing loss were overreported at time of inclusion. This is also congruent with a previous study with the DizzyQuest, in which the reported frequency of vertigo attacks was much higher in a retrospective end of day questionnaire than in the questionnaires which needed to be completed right after a vertigo attack [26]. The lack of PTA variability might have underestimated the relationship between PTA and self-reported hearing loss and tinnitus severity, as a result of the “restriction of range effect”. Future research should therefore try to include patients with many PTA fluctuations during the study period, to more reliably analyse relationships between PTA and symptom severities.

Ears that corresponded with the audiometric criteria of MD (affected ears) showed on average higher self-reported scores with respect to hearing loss and tinnitus severity. This was expected, since the inclusion criteria for MD include hearing loss and tinnitus. Therefore, a selection bias was most likely present. Nevertheless, this shows that the DizzyQuest is able to measure these aspects with significant differences compared to unaffected ears, demonstrating its validity in this context.

Finally, the good correlation between the iPad-based hearing test results and results from “conventional” audiometry obtained in audiological centers, strengthens the use of portable hearing tests in future research [3, 13, 27–29]

Taking all these aspects into account, this study demonstrates that technological advances provide the opportunity to perform multiple measurements in daily life setting, including assessment of neuro-otological symptoms using the DizzyQuest, and assessment of objective hearing thresholds using home-based hearing tests. Using these strategies most likely improves reliability (due to increase in data points) and it increases ecological validity of the findings, since results more closely reflect real-life setting. They also highly facilitate assessment of findings on an individual level, improving precision medicine [30]. It is therefore advised to consider the use of these strategies in future neuro-otological research.

### Limitations

Two main limitations were present in this study. Firstly, during home-based audiometry using the iPad-based hearing test, the noise alert mode was off. Therefore it was not known whether background noise was present when patients performed the hearing test, which might have influenced the results of hearing thresholds. Nevertheless, all patients were explicitly instructed (verbally and written) to perform the test in a quiet room.

Secondly, some patients already started with the DizzyQuest and iPad-based hearing test before the intended starting date, which might have affected the study results. However, most of the patients achieved a high number of valid testing days during the study period [31]. This shows that, once patients started the hearing test, they tended to comply with the study period.

## Conclusion

The combination of the DizzyQuest and iPad-based hearing test, facilitated assessment of self-reported hearing loss and tinnitus severity and their relationship with hearing thresholds (PTA 500–4000 Hz), in a daily life setting. Overall, PTAs were not correlated with self-reported severity of hearing loss and tinnitus. On an individual level, no significant relationship was found between individual PTA difference and corresponding difference in self-reported hearing loss or tinnitus severity between the two ears of the same subjects. However, self-reported hearing loss and tinnitus severity scores were significantly higher in ears that corresponded with audiometric criteria of MD and self-reported hearing loss and tinnitus were significantly related. This study illustrated that it is important to investigate neuro-otological symptoms on an individual level, using multiple measurements. ESM strategies like the DizzyQuest should therefore be considered in future neuro-otological research.

## Supplementary Information

Below is the link to the electronic supplementary material.

Supplementary file1 (PDF 209 KB)

Supplementary file2 (PDF 384 KB)
