# Supplementary material for: The DizzyQuest: relation between self-reported hearing loss, tinnitus and objective hearing thresholds in patients with Meniere’s disease
Source: J Neurol. 2021 Dec 11;269(10):5239–48. doi: 10.1007/s00415-021-10909-8 (PMC9468103; doi:10.1007/s00415-021-10909-8)
Supplement: Supplementary file 1 — Supplementary file1 (PDF 209 KB) [file 415_2021_10909_MOESM1_ESM.pdf]

The DizzyQuest: relation between self-reported hearing loss, tinnitus and objective hearing thresholds in patients with Meniere's disease

Journal of Neurology

E.C. Martin[1], R. Verkaik[1], J.J.A. Stultiens[1], M.R. van de Berg[1], A.M.L. Janssen[2], C. Leue[3], P. Delespaul[3], F. Peeters[4], J. Widdershoven[1], A. Erdkamp[5], S.C.F. van de Weijer[5], H. Blom[6], A. Zwergal[7], E. Grill[8], N. Guinand[9], A. Perez-Fornos[9], D. Tse[10]*, R. van de Berg[1,11]*

Corresponding author: R. van de Berg, raymond.vande.berg@mumc.nl

[1] Division of Balance Disorders, Department of Otorhinolaryngology and Head and Neck Surgery, Maastricht University Medical Center, Maastricht, The Netherlands
[2] Department of Methodology and Statistics, Maastricht University Medical Center, Maastricht, The Netherlands
[3] Department of Psychiatry and Neuropsychology, School for Mental Health and Neuroscience, Maastricht University Medical Center, Maastricht, The Netherlands
[4] Department of Clinical Psychological Science, Faculty of Psychology and Neuroscience, Maastricht University, Maastricht, The Netherlands
[5] mHealth, Maastricht University Medical Center, Maastricht, The Netherlands
[6] Department of Otolaryngology - Head and Neck Surgery, HagaZiekenhuis, the Hague, The Netherlands
[7] Department of Neurology, Ludwig-Maximilians-University of Munich, Munich, Germany.
[8] Department of Medical Informatics, Ludwig-Maximilians-University of Munich, Munich, Germany
[9] Service of Otorhinolaryngology - Head and Neck Surgery, Department of Clinical Neurosciences, Geneva University Hospitals, Geneva, Switzerland.
[10] Department of Otolaryngology - Head & Neck Surgery, University of Ottawa, The Ottawa Hospital, Civic Campus, Ottawa, Canada
[11] Faculty of Physics, Tomsk State Research University, Tomsk, Russia

Keywords: DizzyQuest, Meniere's Disease, Tinnitus, Hearing Loss, Audiometry, Experience Sampling

Evening Questionnaire

Question	Scoring system	Answer options
This was an ordinary day	Likert scale	1 = Not at all<br>4 = Moderate<br>7 = Very
I generally felt well today	Likert scale	1 = Not at all<br>4 = Moderate<br>7 = Very
I generally felt tired today	Likert scale	1 = Not at all<br>4 = Moderate<br>7 = Very
I generally felt relaxed today	Likert scale	1 = Not at all<br>4 = Moderate<br>7 = Very
I generally worried a lot today	Likert scale	1 = Not at all<br>4 = Moderate<br>7 = Very
I generally was able to concentrate well today	Likert scale	1 = Not at all<br>4 = Moderate<br>7 = Very
I felt dizzy today	Likert scale	1 = Not at all<br>4 = Moderate<br>7 = Very
I felt nauseous today	Likert scale	1 = Not at all<br>4 = Moderate<br>7 = Very
I had balance issues today	Likert scale	1 = Not at all<br>4 = Moderate<br>7 = Very
Today I suffered from hearing loss: left	Likert scale	1 = Not at all<br>4 = Moderate<br>7 = Very
Today I suffered from hearing loss: right	Likert scale	1 = Not at all<br>4 = Moderate<br>7 = Very
Today I suffered from tinnitus: left	Likert scale	1 = Not at all<br>4 = Moderate<br>7 = Very
Today I suffered from tinnitus: right	Likert scale	1 = Not at all<br>4 = Moderate<br>7 = Very
Today I suffered from aural fullness	Likert scale	1 = Not at all<br>4 = Moderate<br>7 = Very
Today I suffered from headache	Likert scale	1 = Not at all<br>4 = Moderate<br>7 = Very
Today I suffered from light-sensitivity	Likert scale	1 = Not at all<br>4 = Moderate<br>7 = Very

Today I suffered from sound-sensitivity	Likert scale	1 = Not at all<br>4 = Moderate<br>7 = Very
Today I suffered from light flashes and/or zigzag lines	Likert scale	1 = Not at all<br>4 = Moderate<br>7 = Very
Today I suffered from numbness or tingling in arms, legs and/or face	Likert scale	1 = Not at all<br>4 = Moderate<br>7 = Very
Today I suffered from visual problems when moving	Likert scale	1 = Not at all<br>4 = Moderate<br>7 = Very
Today I suffered from difficulties reading	Likert scale	1 = Not at all<br>4 = Moderate<br>7 = Very
How many attacks of dizziness, nausea and/or hearing loss have you had today?	Multiple choice option	- 0<br>- 1<br>- 2<br>- 3 or more
Did the first attack today already start yesterday?	Multiple choice option	- Yes<br>- No
The attacks were provoked by:	Multiple choice option (maximum 5 options)	- Head or body movements<br>- Strong stimuli (visual, sound, busy places)<br>- Stress<br>- Nothing, started spontaneously<br>- Something else:…
If chosen 'Something else:…' What else provoked the attack?	Open answer option (maximum 120 characters)	
I was unable to function properly because of the attacks for:	Multiple choice option	1. <20 minutes<br>2. 20 minutes – 3 hours<br>3. 3 hours – 12 hours<br>4. >12 hours
To what extent were you limited in your activities today?	Likert scale	1 = Not at all<br>4 = Moderate<br>7 = Very
In which type of activities were you limited the most today?	Multiple choice option (maximum 1 option)	1. Work, study<br>2. Household<br>3. Social<br>4. Travelling, on the way<br>5. Hobbies<br>6. Sports<br>7. Sleeping
