# Supplementary material for: The DizzyQuest: relation between self-reported hearing loss, tinnitus and objective hearing thresholds in patients with Meniere’s disease
Source: J Neurol. 2021 Dec 11;269(10):5239–48. doi: 10.1007/s00415-021-10909-8 (PMC9468103; doi:10.1007/s00415-021-10909-8)
Supplement: Supplementary file 2 — Supplementary file2 (PDF 384 KB) [file 415_2021_10909_MOESM2_ESM.pdf]

The DizzyQuest: relation between self-reported hearing loss, tinnitus and objective hearing thresholds in patients with Meniere's disease

Journal of Neurology

E.C. Martin[1], R. Verkaik[1], J.J.A. Stultiens[1], M.R. van de Berg[1], A.M.L. Janssen[2], C. Leue[3], P. Delespaul[3], F. Peeters[4], J. Widdershoven[1], A. Erdkamp[5], S.C.F. van de Weijer[5], H. Blom[6], A. Zwergal[7], E. Grill[8], N. Guinand[9], A. Perez-Fornos[9], D. Tse[10]*, R. van de Berg[1,11]*

Corresponding author: R. van de Berg, raymond.vande.berg@mumc.nl

[1] Division of Balance Disorders, Department of Otorhinolaryngology and Head and Neck Surgery, Maastricht University Medical Center, Maastricht, The Netherlands
[2] Department of Methodology and Statistics, Maastricht University Medical Center, Maastricht, The Netherlands
[3] Department of Psychiatry and Neuropsychology, School for Mental Health and Neuroscience, Maastricht University Medical Center, Maastricht, The Netherlands
[4] Department of Clinical Psychological Science, Faculty of Psychology and Neuroscience, Maastricht University, Maastricht, The Netherlands
[5] mHealth, Maastricht University Medical Center, Maastricht, The Netherlands
[6] Department of Otolaryngology - Head and Neck Surgery, HagaZiekenhuis, the Hague, The Netherlands
[7] Department of Neurology, Ludwig-Maximilians-University of Munich, Munich, Germany.
[8]Department of Medical Informatics, Ludwig-Maximilians-University of Munich, Munich, Germany
[9] Service of Otorhinolaryngology - Head and Neck Surgery, Department of Clinical Neurosciences, Geneva University Hospitals, Geneva, Switzerland.
[10] Department of Otolaryngology - Head & Neck Surgery, University of Ottawa, The Ottawa Hospital, Civic Campus, Ottawa, Canada
[11] Faculty of Physics, Tomsk State Research University, Tomsk, Russia
Keywords: DizzyQuest, Meniere's Disease, Tinnitus, Hearing Loss, Audiometry, Experience Sampling

Audiometric iPad-based hearing test results of 15 patients over 21 study days. Horizontal axis indicates the frequencies ranging from 250 to 8000Hz. Vertical axis indicates the hearing thresholds in dB. The colors indicate the individual study days. AD = right ear. AS = left ear.

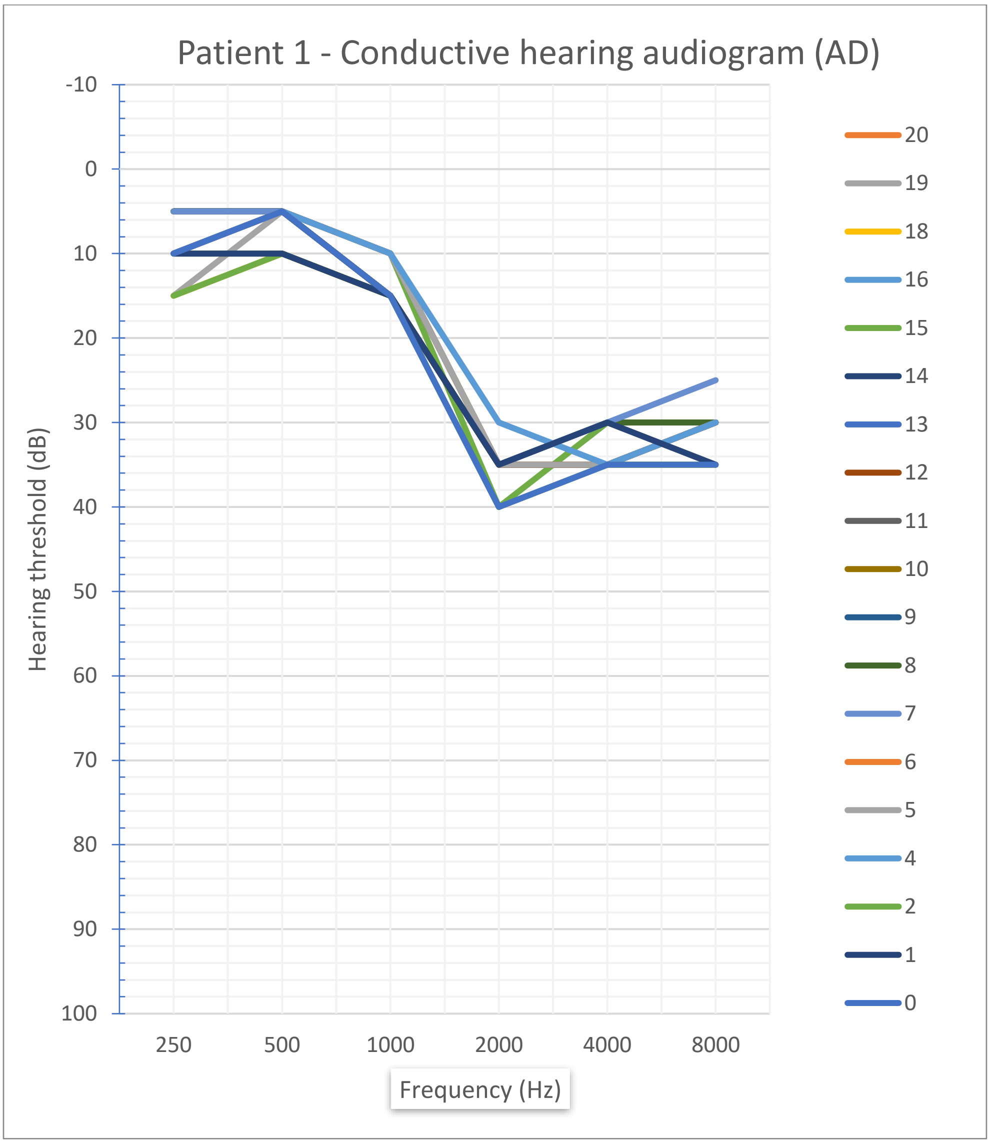

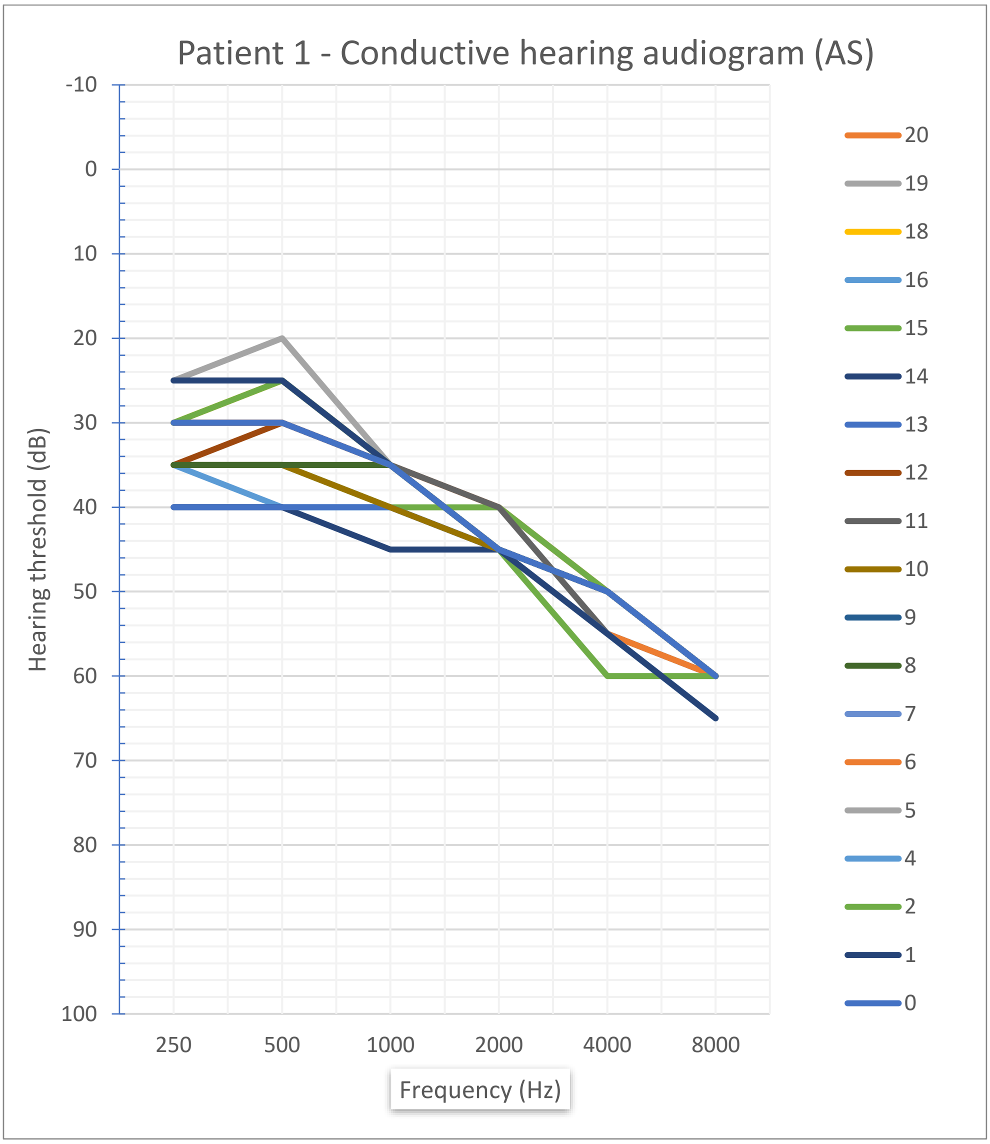
Patient 1 - Conductive hearing audiogram (AS)
-10
0
10
20
30
40
50
60
70
80
90
100
Hearing threshold (dB)
250
500
1000
2000
4000
8000
Frequency (Hz)
20
19
18
16
15
14
13
12
11
10
9
8
7
6
5
4
2
1
0

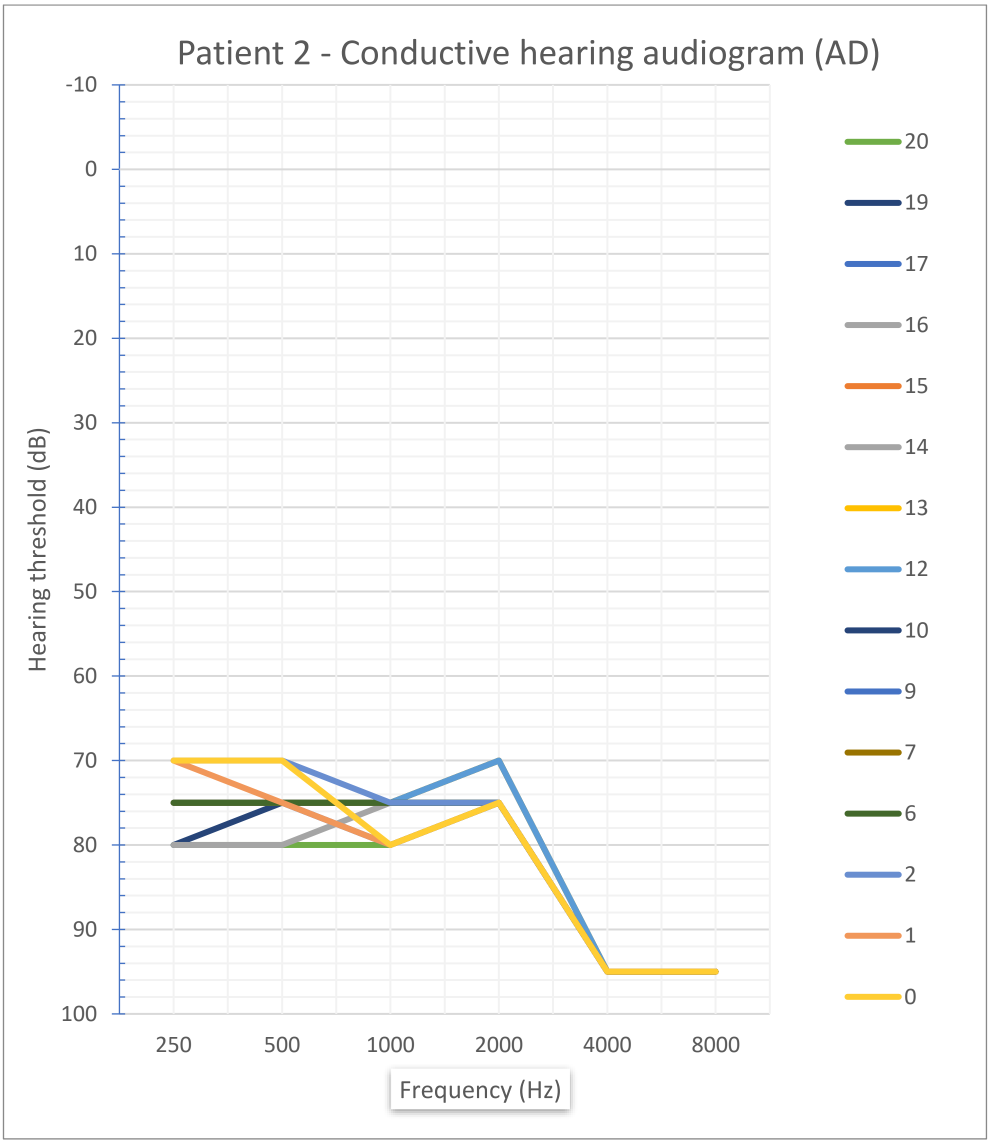
Patient 2 - Conductive hearing audiogram (AD)
Hearing threshold (dB)
-10
0
10
20
30
40
50
60
70
80
90
100
250
500
1000
2000
4000
8000
Frequency (Hz)
20
19
17
16
15
14
13
12
10
9
7
6
2
1
0

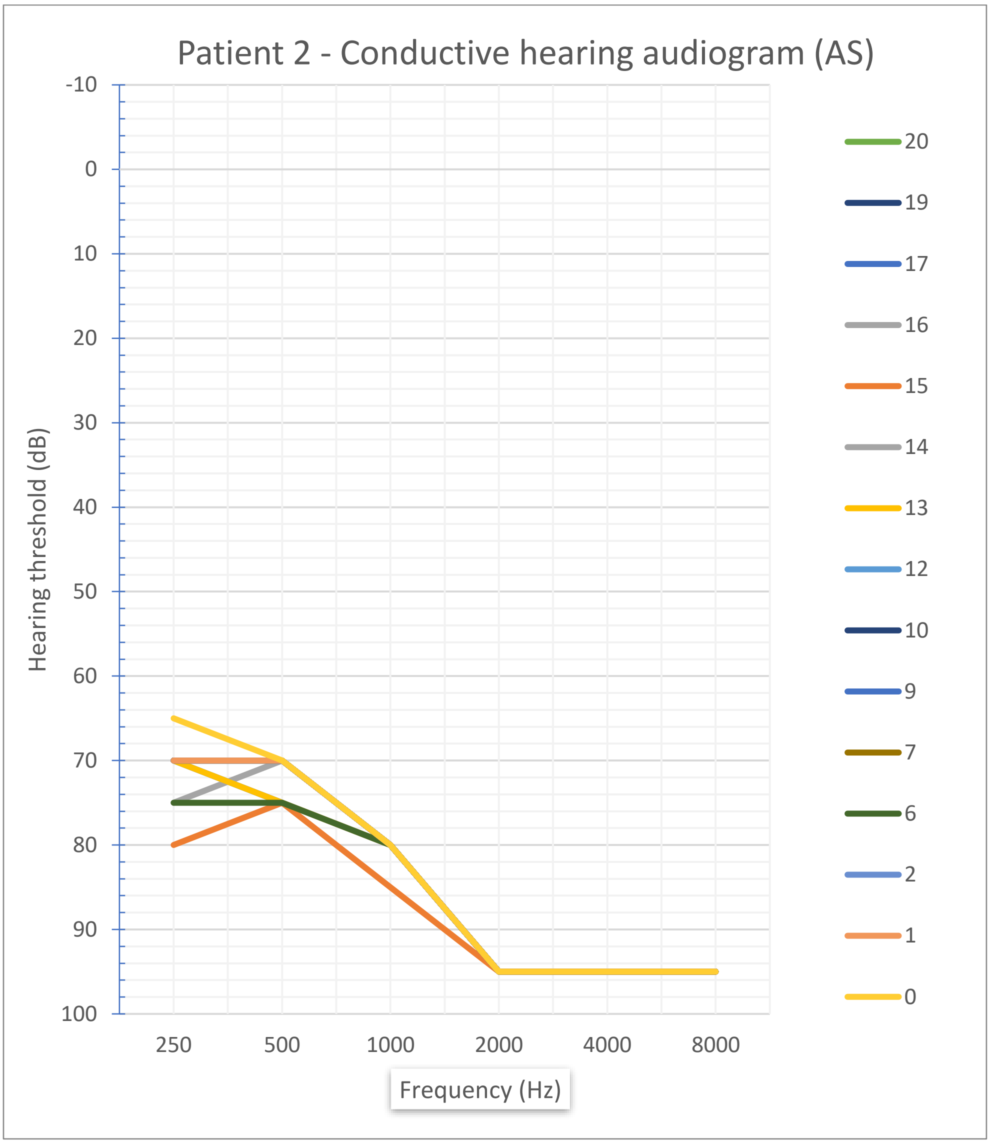
Patient 2 - Conductive hearing audiogram (AS)
Hearing threshold (dB)
-10
0
10
20
30
40
50
60
70
80
90
100
250
500
1000
2000
4000
8000
Frequency (Hz)
20
19
17
16
15
14
13
12
10
9
7
6
2
1
0

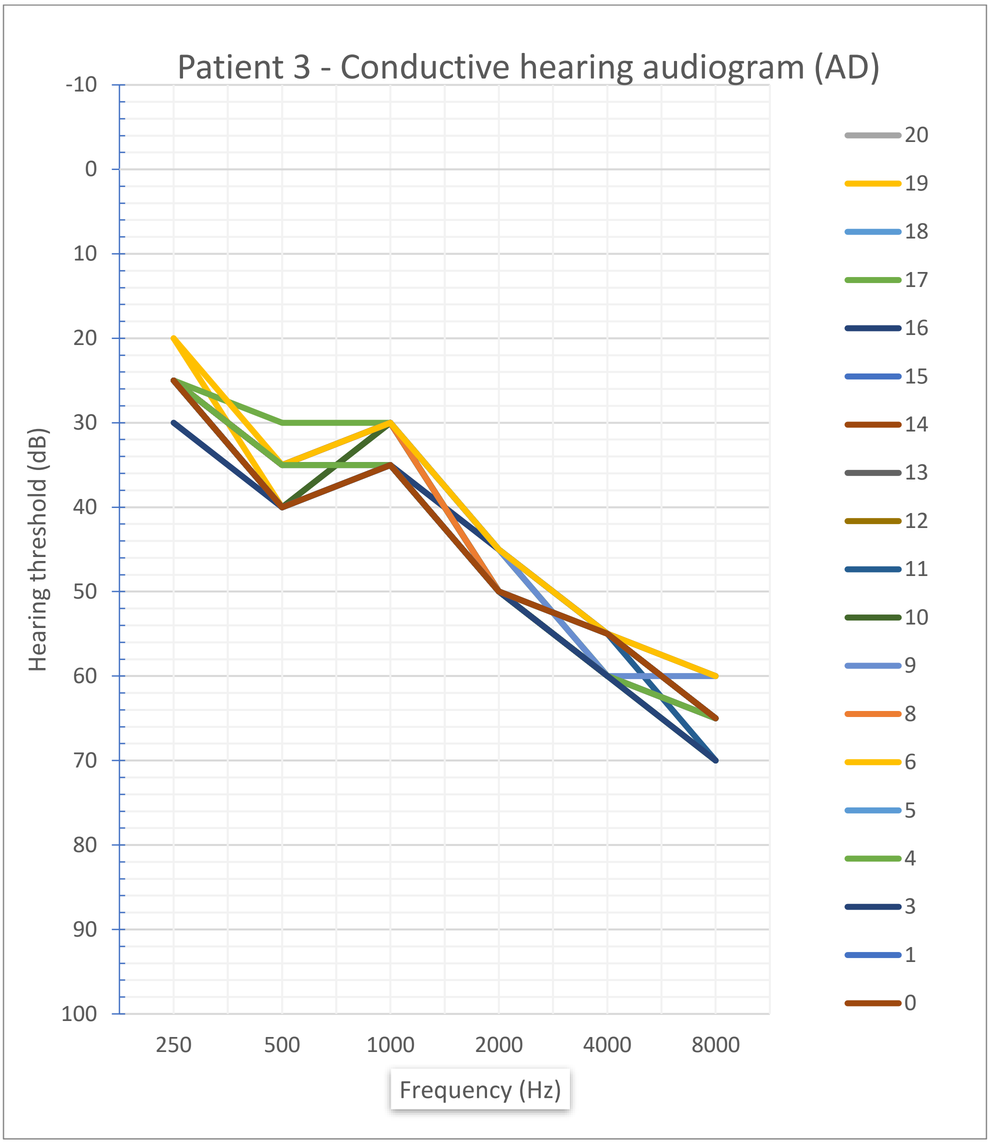
Patient 3 - Conductive hearing audiogram (AD)
Hearing threshold (dB)
-10
0
10
20
30
40
50
60
70
80
90
100
250
500
1000
2000
4000
8000
Frequency (Hz)
20
19
18
17
16
15
14
13
12
11
10
9
8
6
5
4
3
1
0

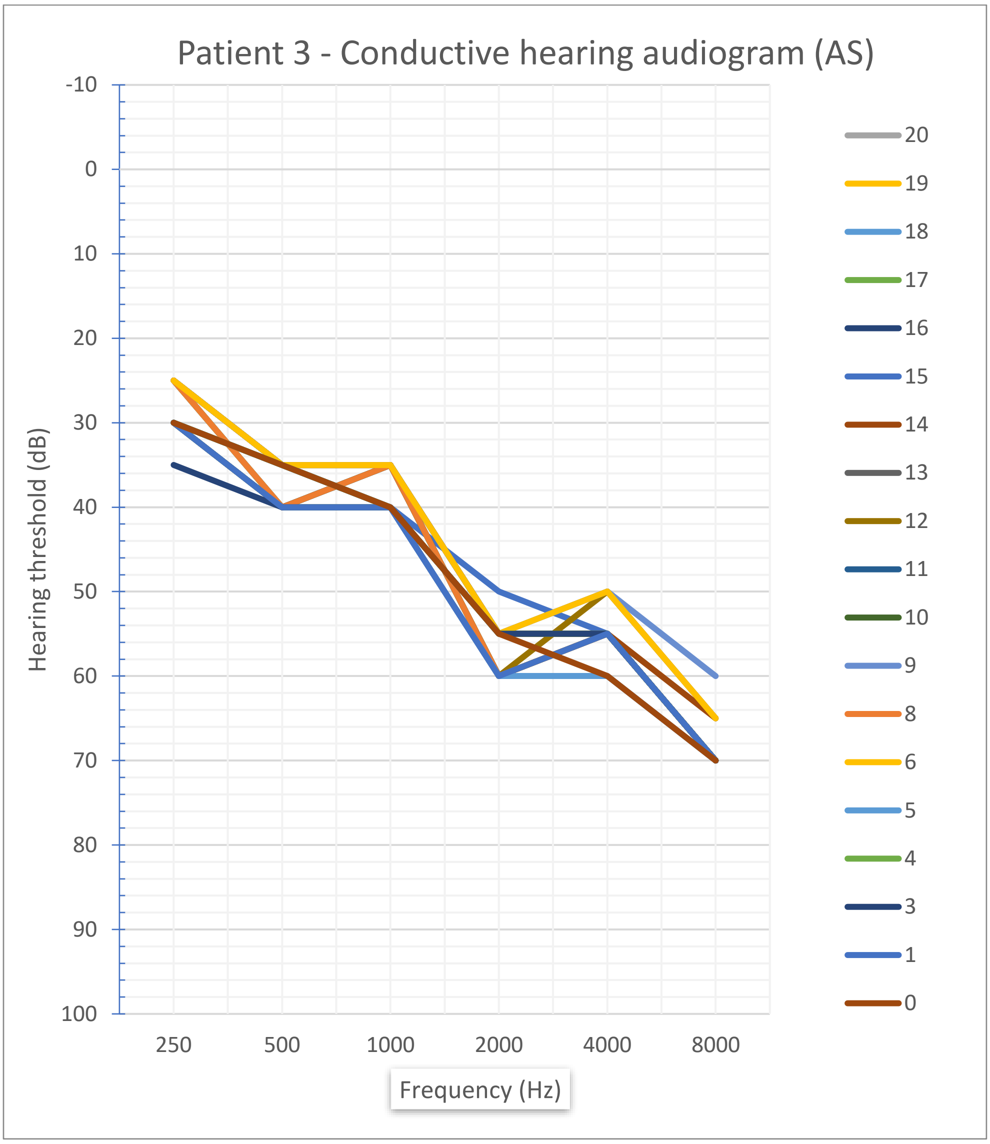

Patient 3 - Conductive hearing audiogram (AS)
Hearing threshold (dB)
-10
0
10
20
30
40
50
60
70
80
90
100
250
500
1000
2000
4000
8000
Frequency (Hz)
20
19
18
17
16
15
14
13
12
11
10
9
8
6
5
4
3
1
0

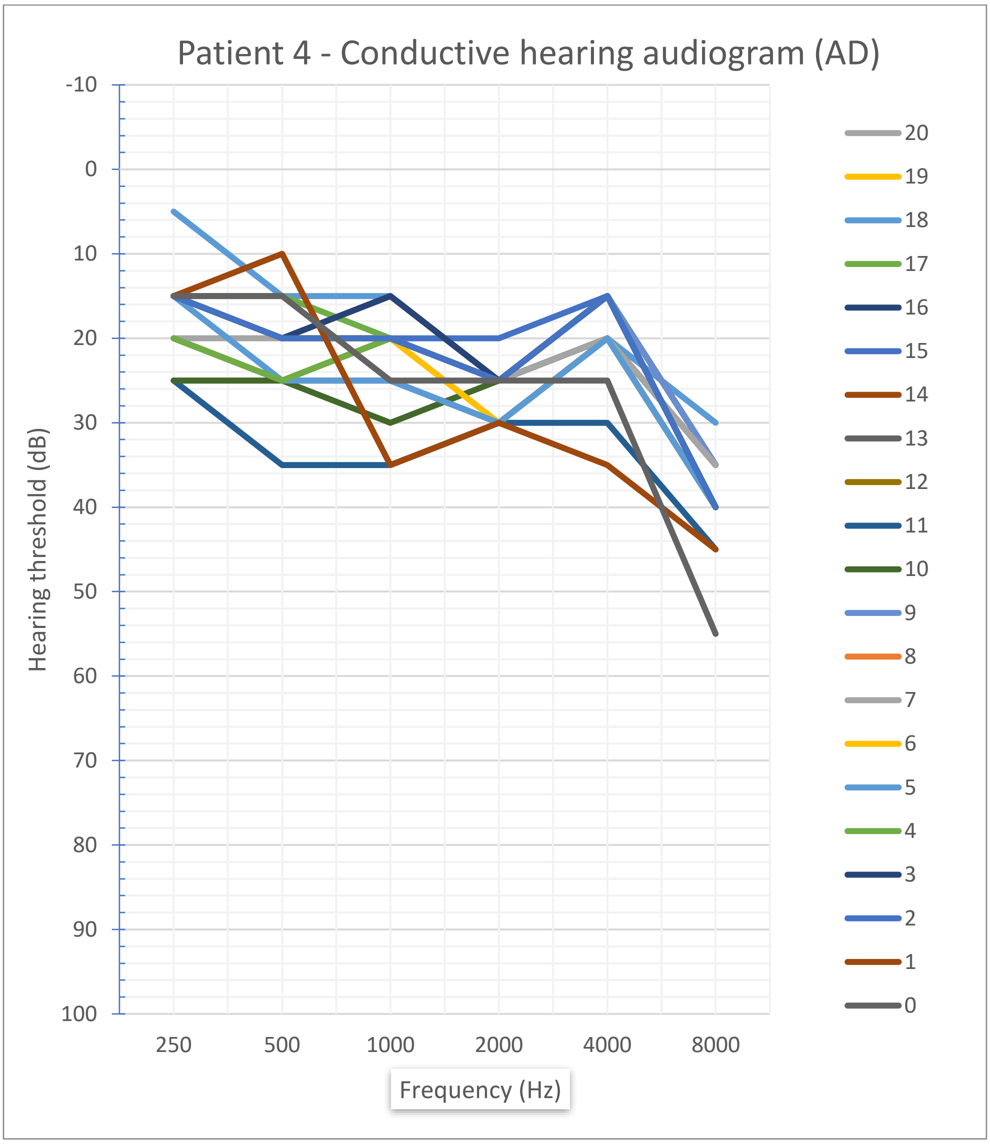

Patient 4 - Conductive hearing audiogram (AD)
Hearing threshold (dB)
-10
0
10
20
30
40
50
60
70
80
90
100
250
500
1000
2000
4000
8000
Frequency (Hz)
20
19
18
17
16
15
14
13
12
11
10
9
8
7
6
5
4
3
2
1
0

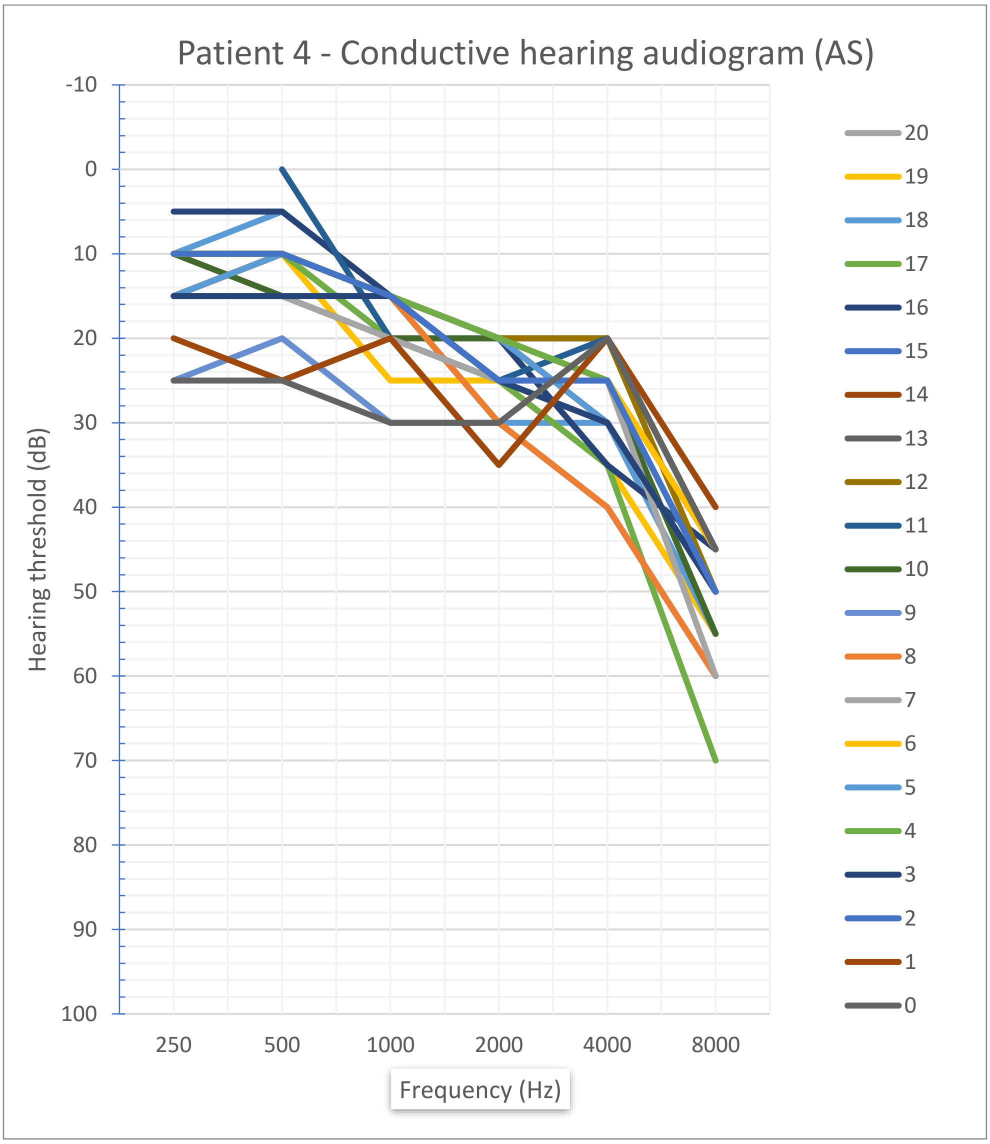

Patient 4 - Conductive hearing audiogram (AS)
-10
0
10
20
30
40
50
60
70
80
90
100
Hearing threshold (dB)
250
500
1000
2000
4000
8000
Frequency (Hz)
20
19
18
17
16
15
14
13
12
11
10
9
8
7
6
5
4
3
2
1
0

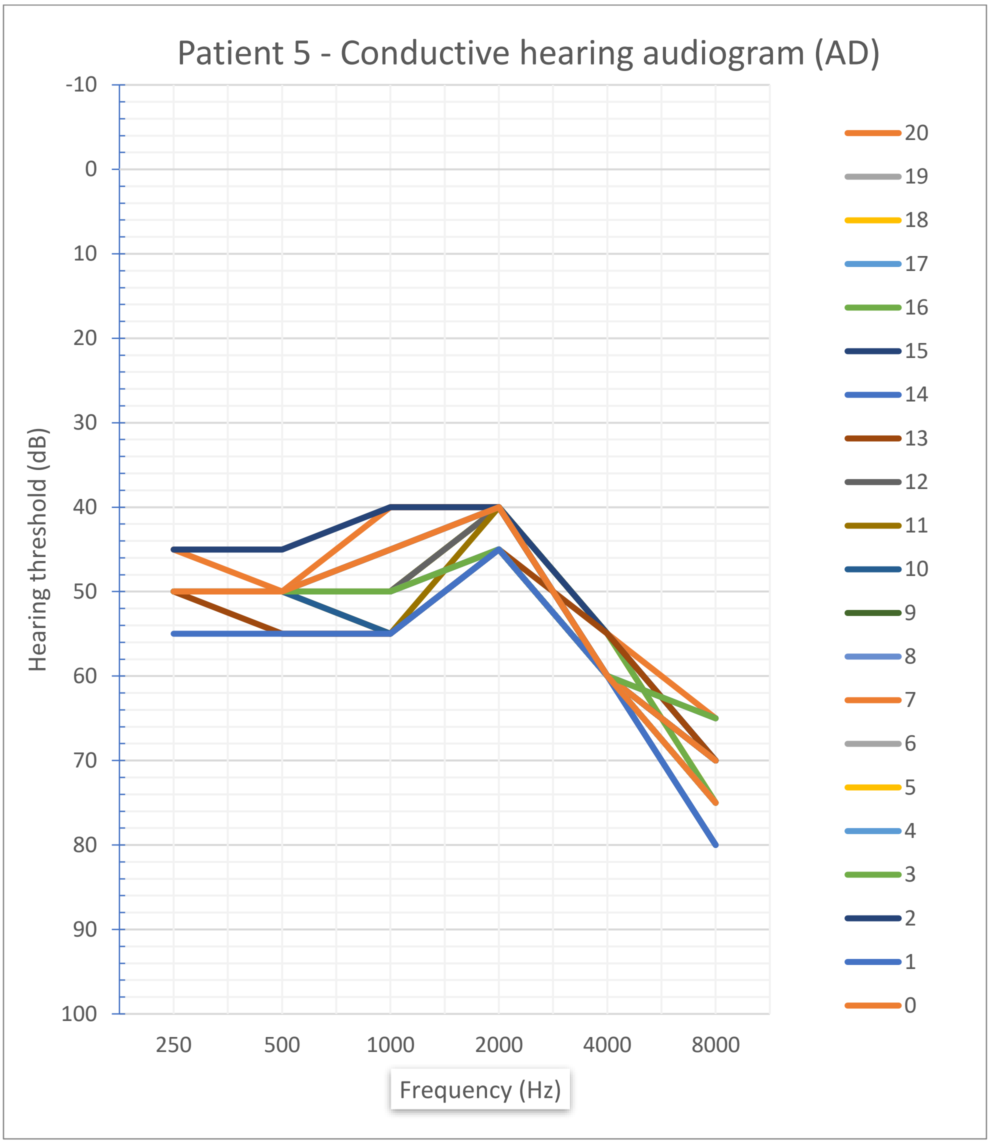

Patient 5 - Conductive hearing audiogram (AD)
Hearing threshold (dB)
-10
0
10
20
30
40
50
60
70
80
90
100
250
500
1000
2000
4000
8000
Frequency (Hz)
20
19
18
17
16
15
14
13
12
11
10
9
8
7
6
5
4
3
2
1
0

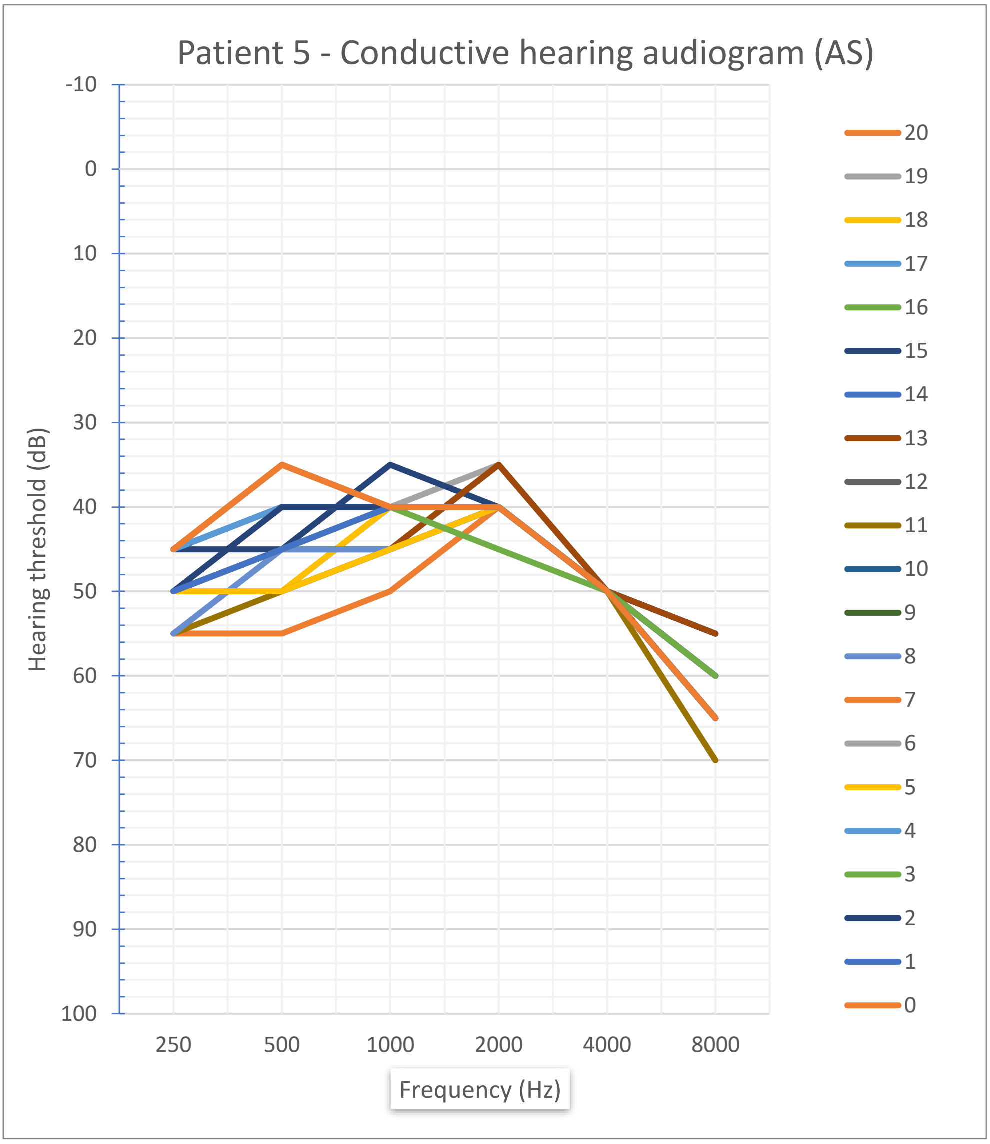

Patient 5 - Conductive hearing audiogram (AS)
Hearing threshold (dB)
-10
0
10
20
30
40
50
60
70
80
90
100
250
500
1000
2000
4000
8000
Frequency (Hz)
20
19
18
17
16
15
14
13
12
11
10
9
8
7
6
5
4
3
2
1
0

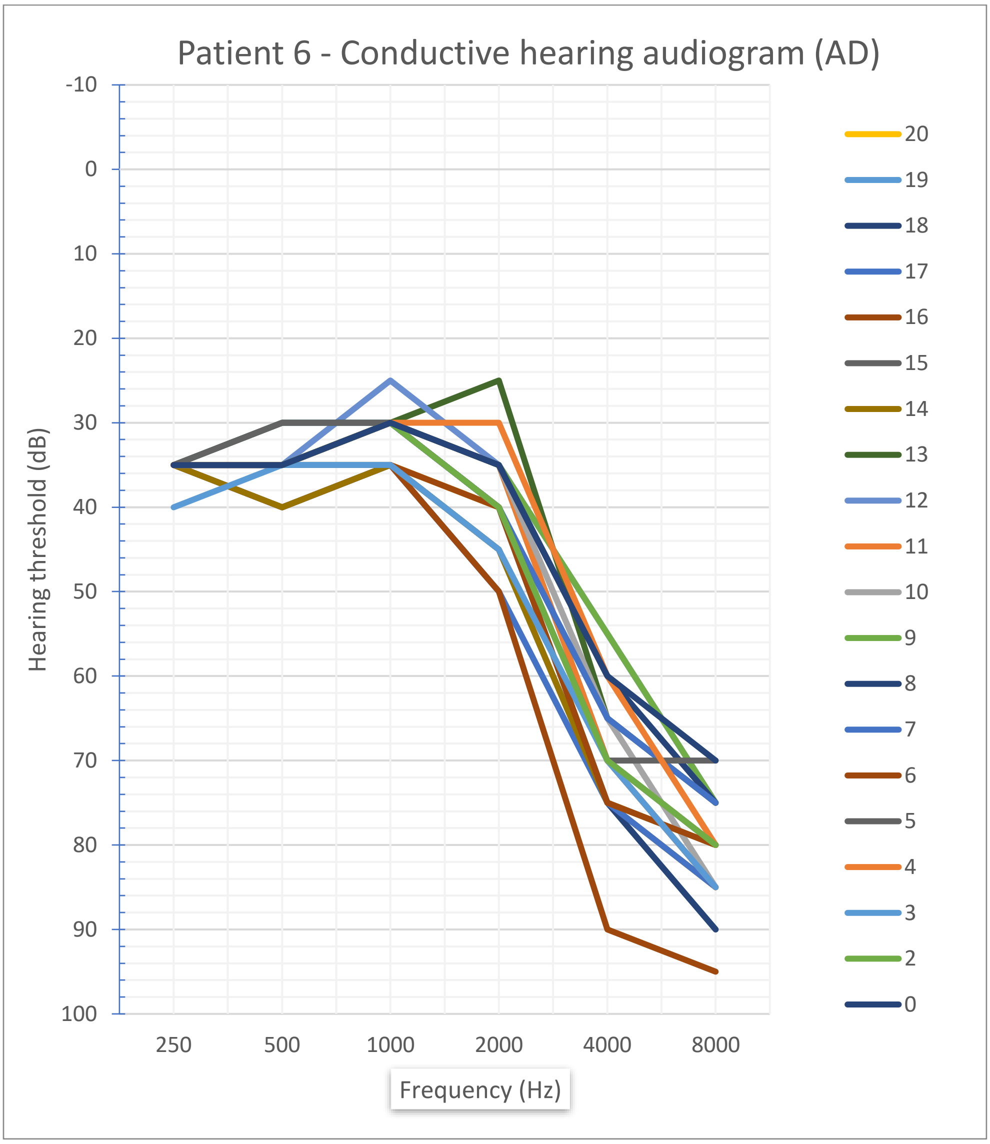
Patient 6 - Conductive hearing audiogram (AD)
-10
0
10
20
30
40
50
60
70
80
90
100
Hearing threshold (dB)
250
500
1000
2000
4000
8000
Frequency (Hz)
20
19
18
17
16
15
14
13
12
11
10
9
8
7
6
5
4
3
2
0

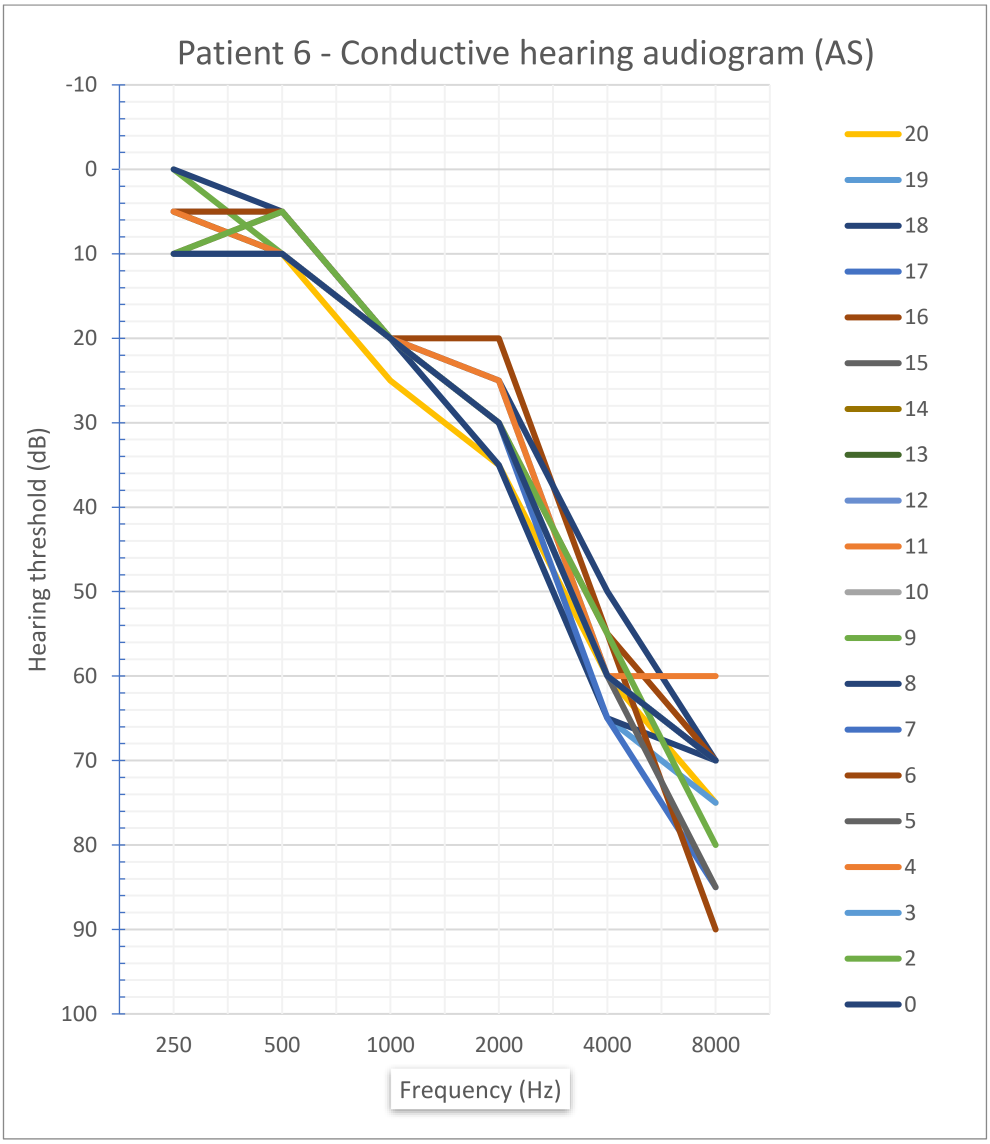

Patient 6 - Conductive hearing audiogram (AS)
-10
0
10
20
30
40
50
60
70
80
90
100
Hearing threshold (dB)
250
500
1000
2000
4000
8000
Frequency (Hz)
20
19
18
17
16
15
14
13
12
11
10
9
8
7
6
5
4
3
2
0

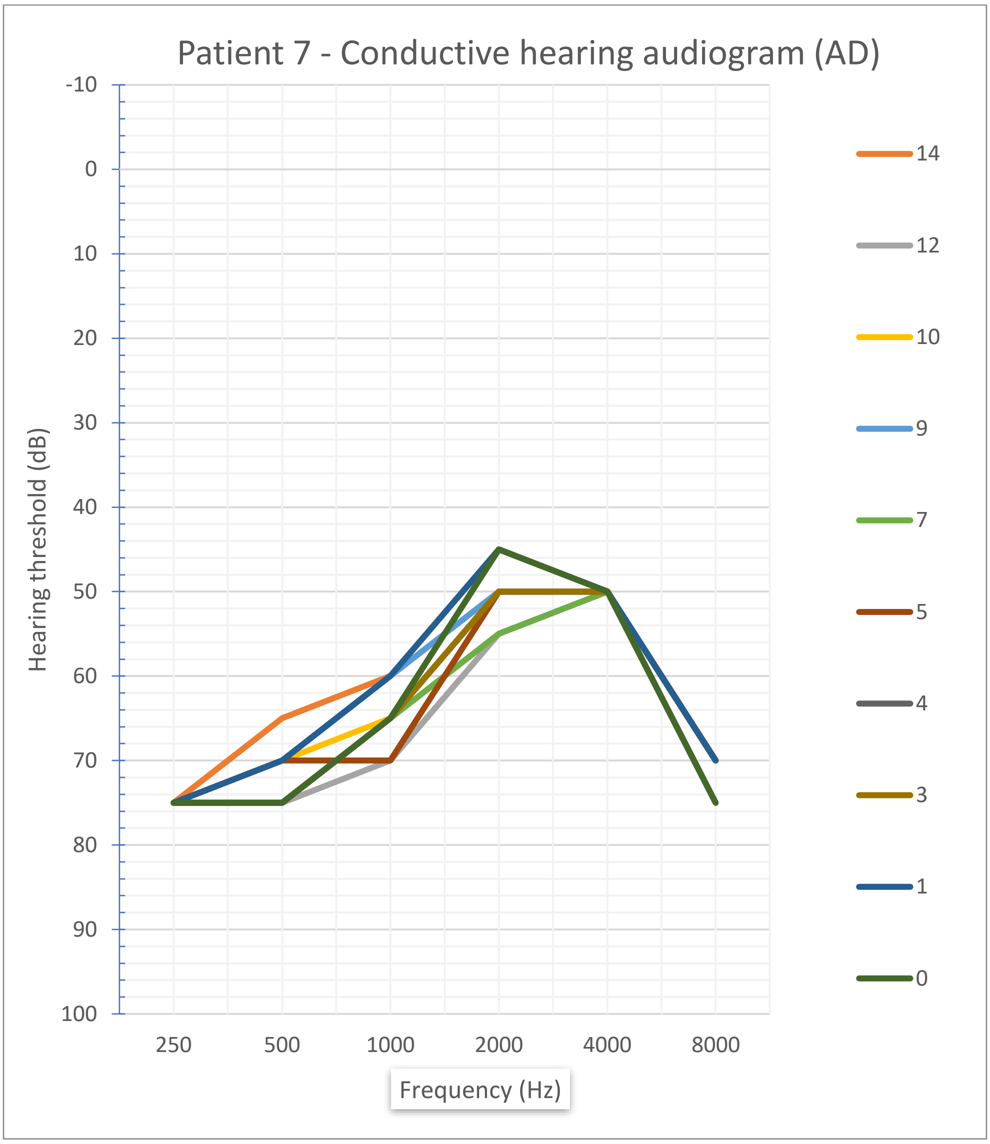

Patient 7 - Conductive hearing audiogram (AD)
-10
0
10
20
30
40
50
60
70
80
90
100
Hearing threshold (dB)
250
500
1000
2000
4000
8000
Frequency (Hz)
14
12
10
9
7
5
4
3
1
0

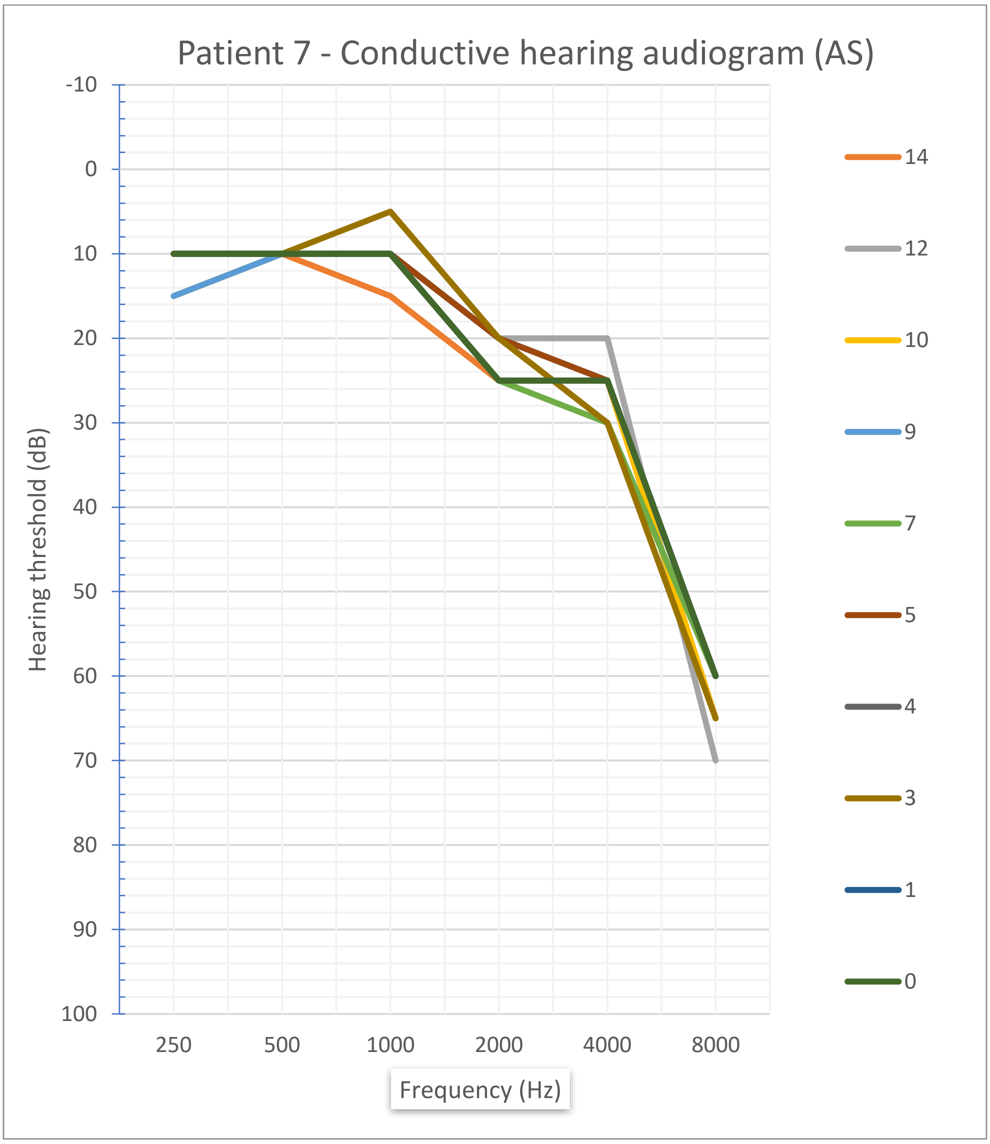

Patient 7 - Conductive hearing audiogram (AS)
Hearing threshold (dB)
-10
0
10
20
30
40
50
60
70
80
90
100
250
500
1000
2000
4000
8000
Frequency (Hz)
14
12
10
9
7
5
4
3
1
0

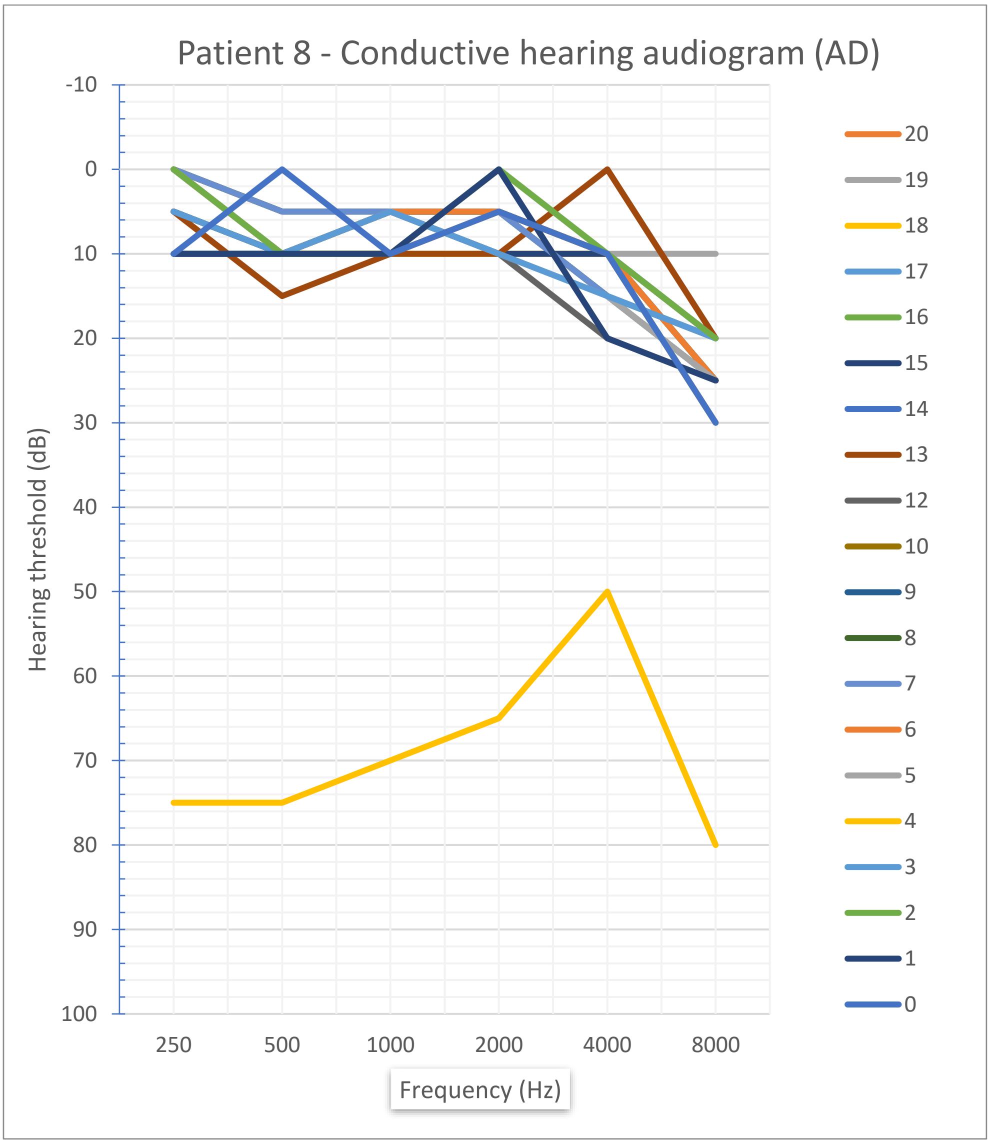

Patient 8 - Conductive hearing audiogram (AD)
Hearing threshold (dB)
-10
0
10
20
30
40
50
60
70
80
90
100
250
500
1000
2000
4000
8000
Frequency (Hz)
20
19
18
17
16
15
14
13
12
10
9
8
7
6
5
4
3
2
1
0

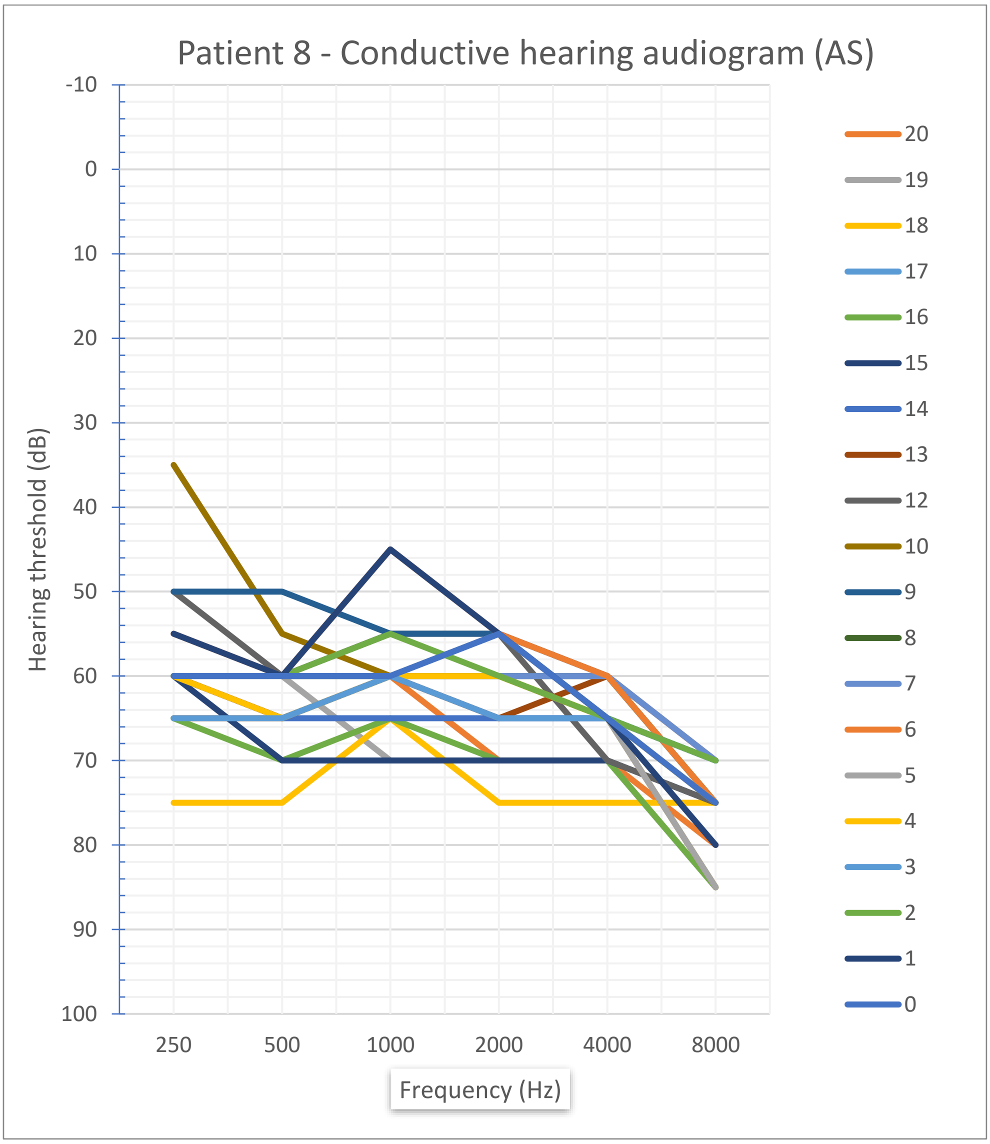

Patient 8 - Conductive hearing audiogram (AS)
Hearing threshold (dB)
-10
0
10
20
30
40
50
60
70
80
90
100
250
500
1000
2000
4000
8000
Frequency (Hz)
20
19
18
17
16
15
14
13
12
10
9
8
7
6
5
4
3
2
1
0

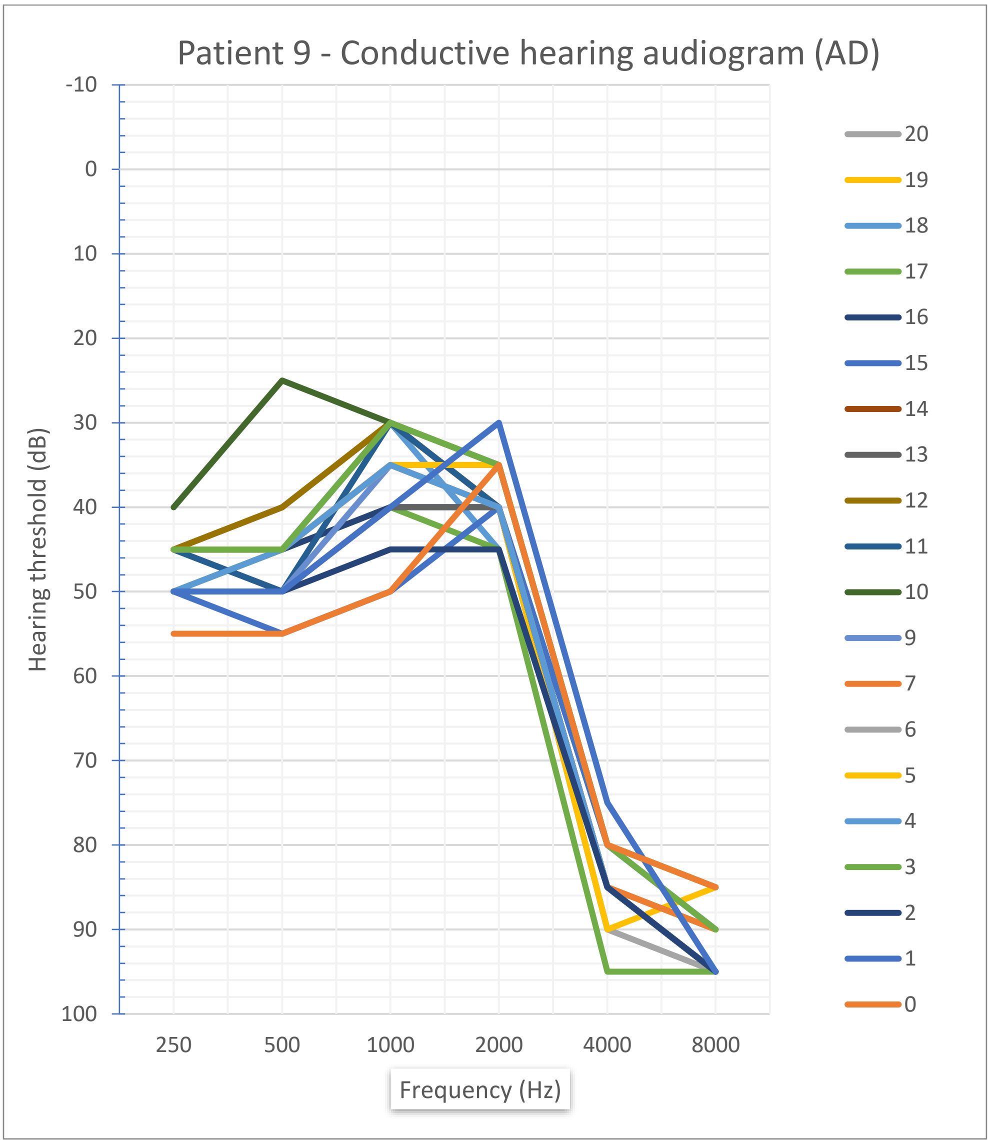

Patient 9 - Conductive hearing audiogram (AD)
Hearing threshold (dB)
-10
0
10
20
30
40
50
60
70
80
90
100
250
500
1000
2000
4000
8000
Frequency (Hz)
20
19
18
17
16
15
14
13
12
11
10
9
7
6
5
4
3
2
1
0

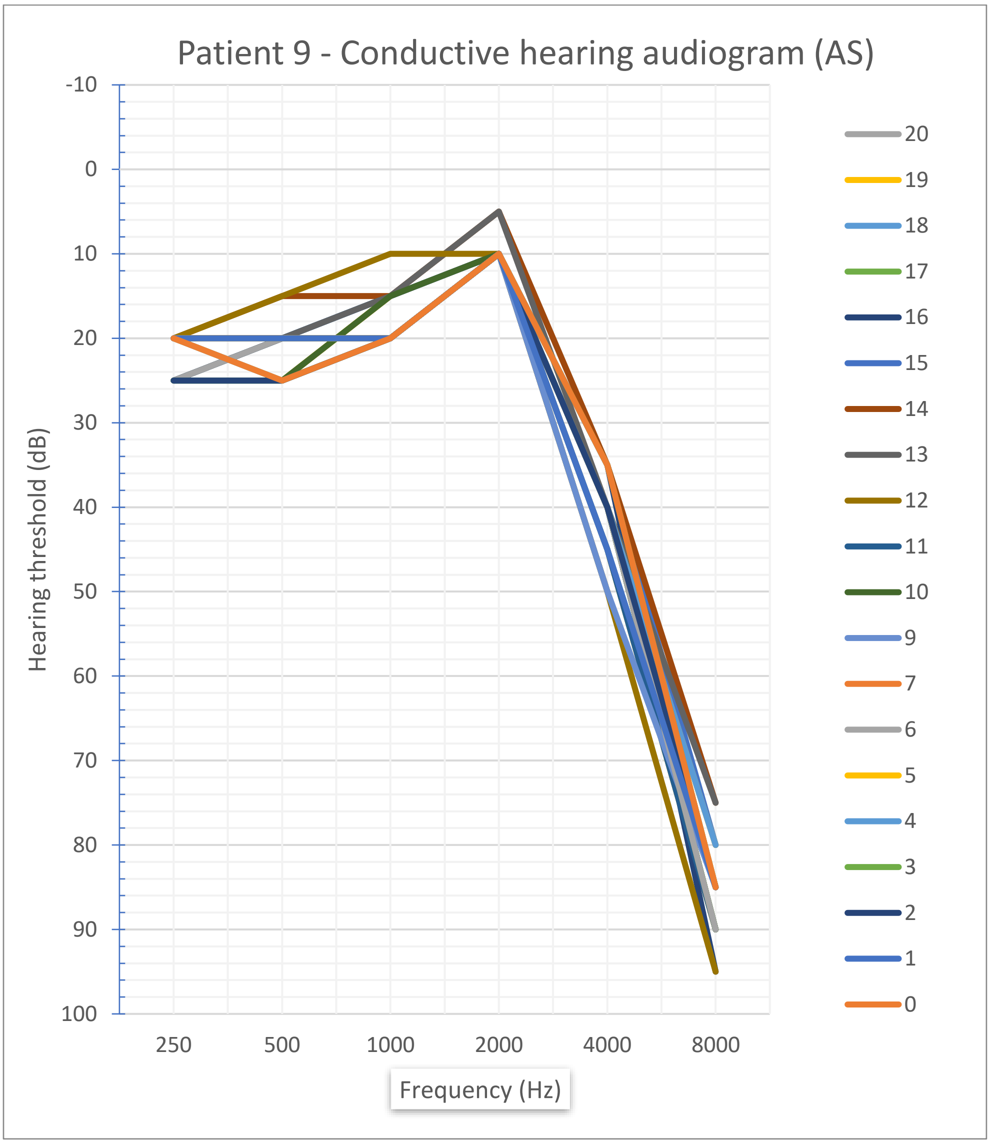
Patient 9 - Conductive hearing audiogram (AS)
Hearing threshold (dB)
-10
0
10
20
30
40
50
60
70
80
90
100
250
500
1000
2000
4000
8000
Frequency (Hz)
20
19
18
17
16
15
14
13
12
11
10
9
7
6
5
4
3
2
1
0

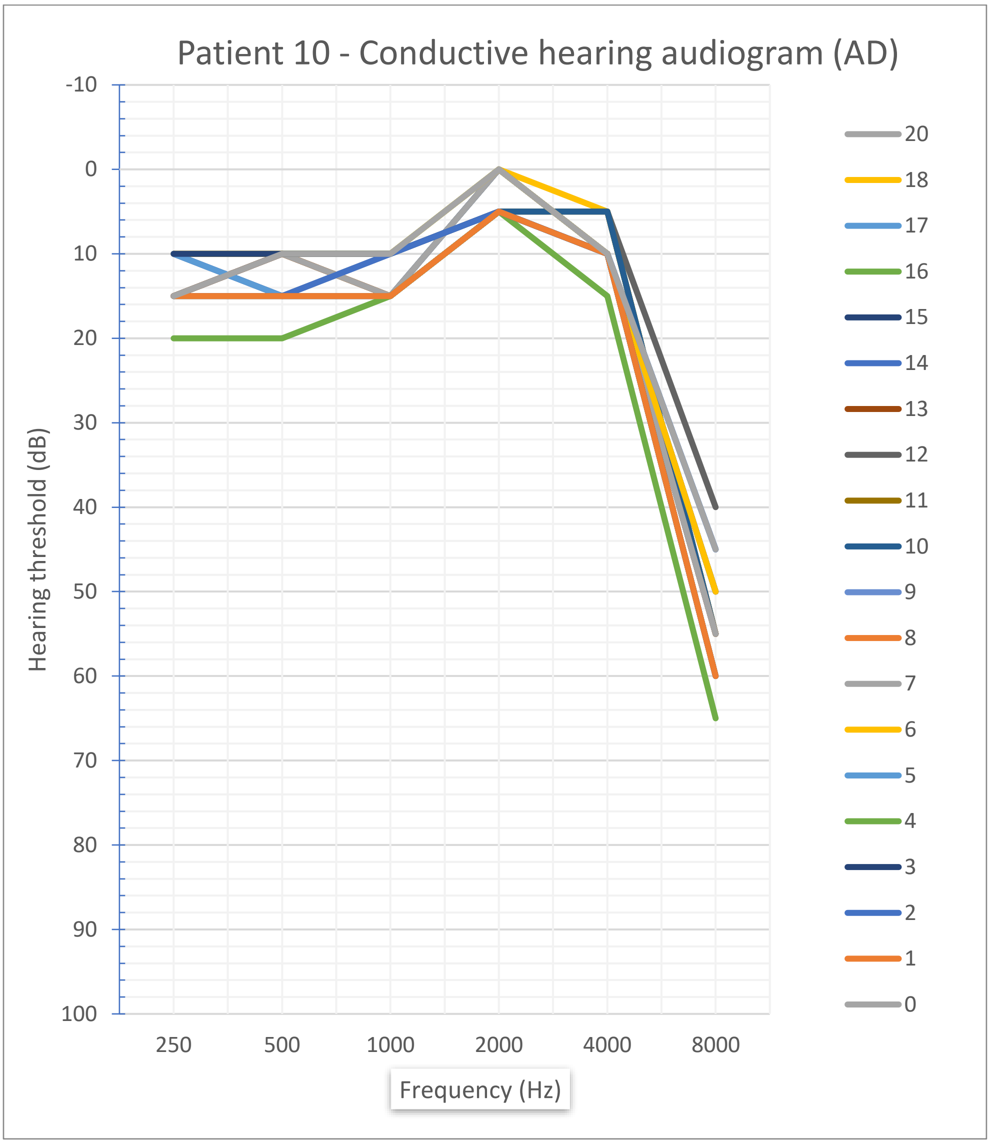
Patient 10 - Conductive hearing audiogram (AD)
Hearing threshold (dB)
-10
0
10
20
30
40
50
60
70
80
90
100
250
500
1000
2000
4000
8000
Frequency (Hz)
20
18
17
16
15
14
13
12
11
10
9
8
7
6
5
4
3
2
1
0

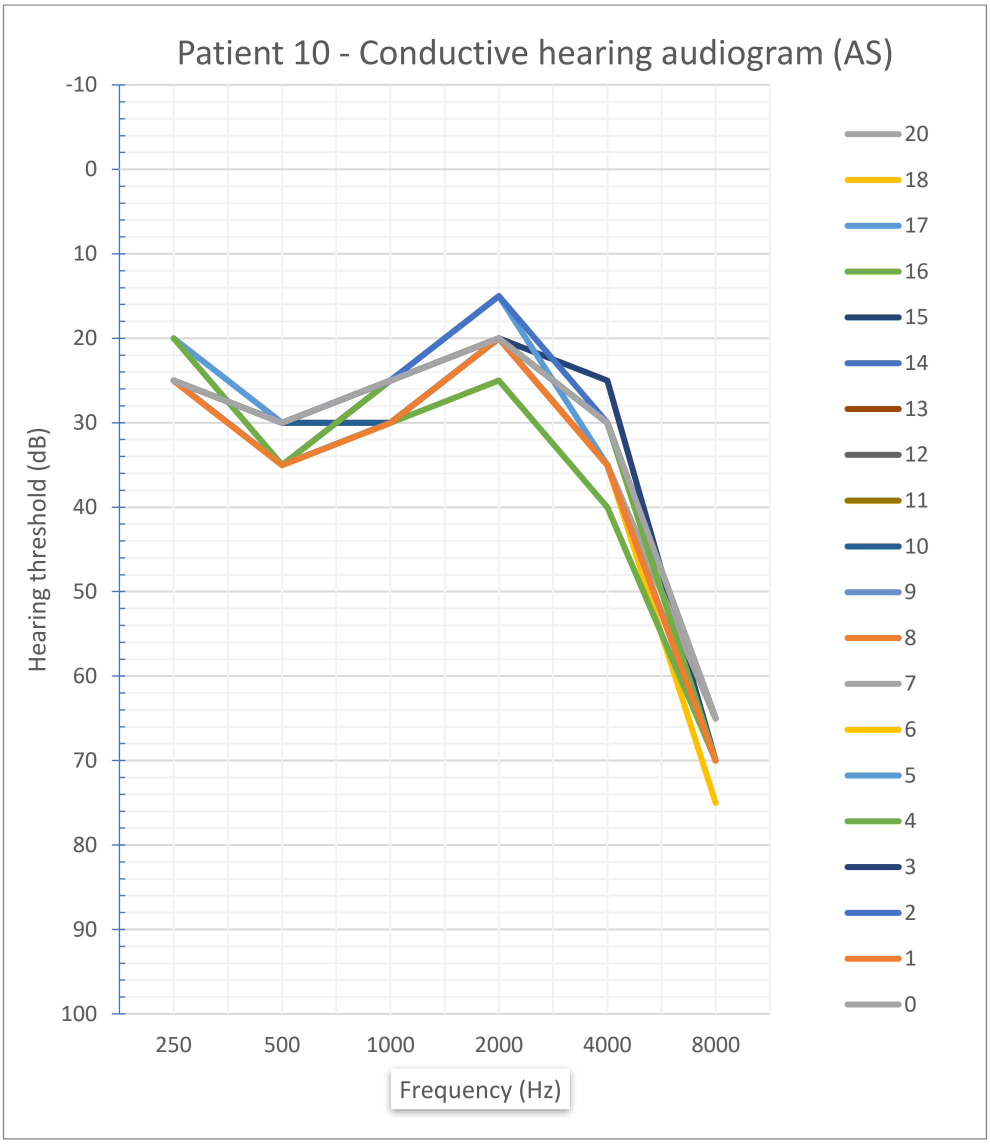

Patient 10 - Conductive hearing audiogram (AS)
Hearing threshold (dB)
-10
0
10
20
30
40
50
60
70
80
90
100
250
500
1000
2000
4000
8000
Frequency (Hz)
20
18
17
16
15
14
13
12
11
10
9
8
7
6
5
4
3
2
1
0

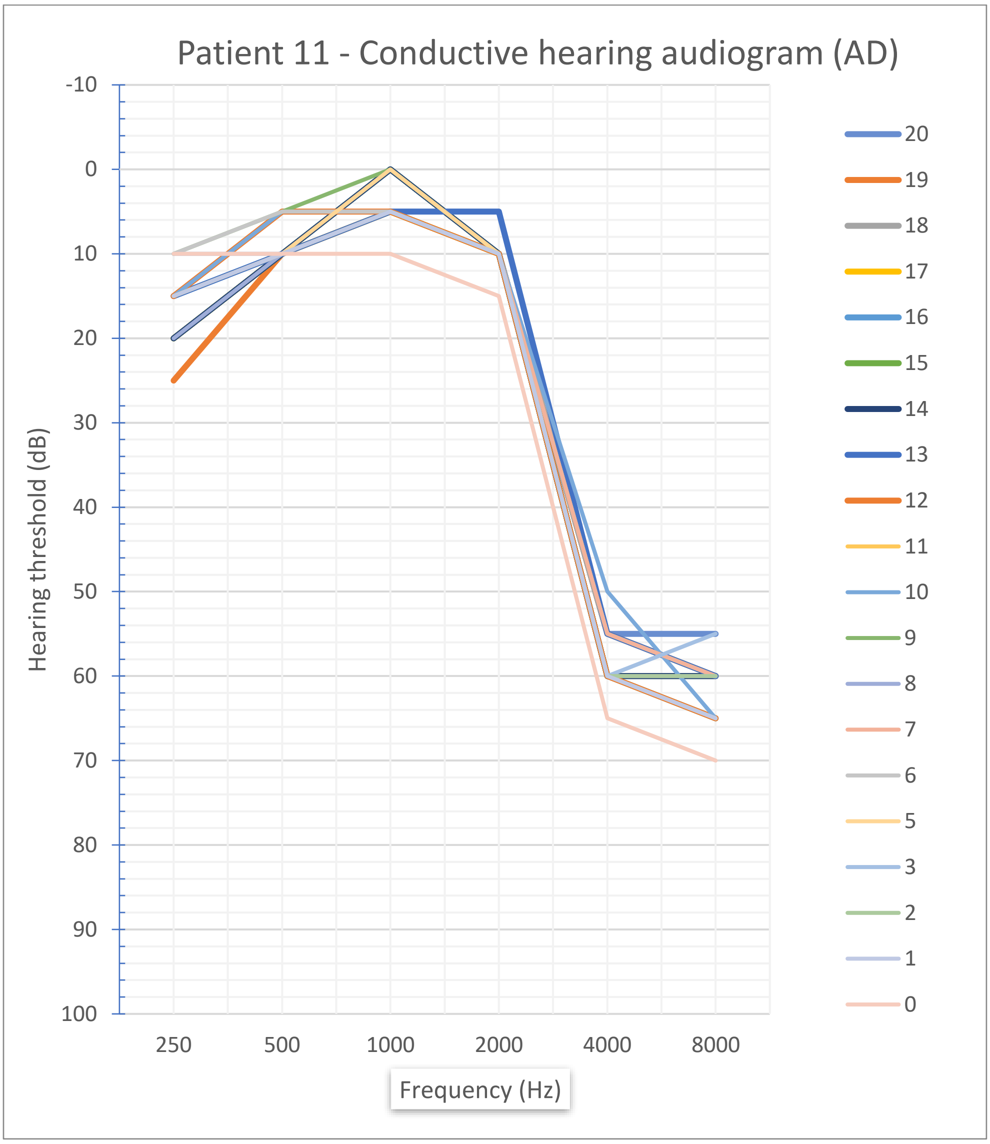

Patient 11 - Conductive hearing audiogram (AD)
Hearing threshold (dB)
-10
0
10
20
30
40
50
60
70
80
90
100
250
500
1000
2000
4000
8000
Frequency (Hz)
20
19
18
17
16
15
14
13
12
11
10
9
8
7
6
5
3
2
1
0

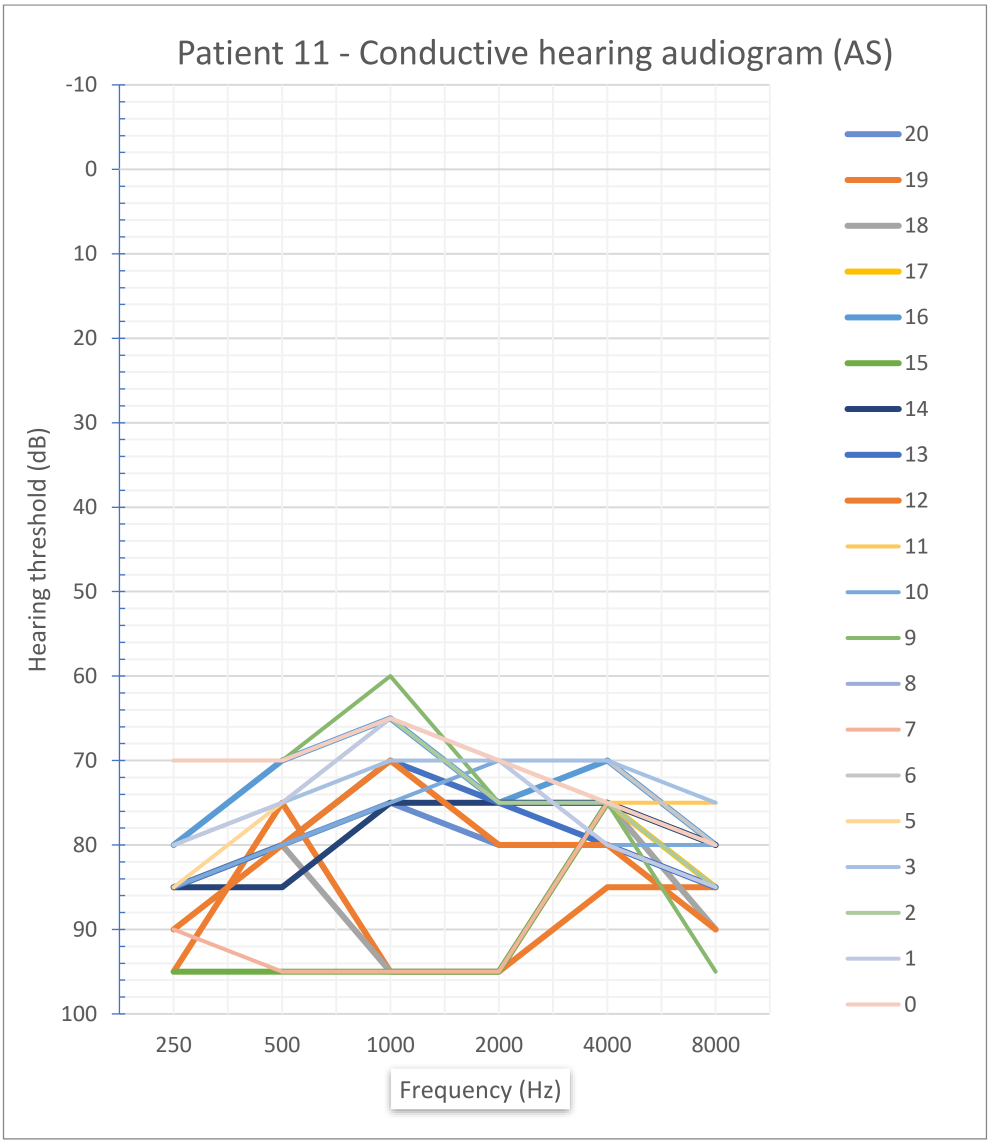

Patient 11 - Conductive hearing audiogram (AS)
Hearing threshold (dB)
-10
0
10
20
30
40
50
60
70
80
90
100
250
500
1000
2000
4000
8000
Frequency (Hz)
20
19
18
17
16
15
14
13
12
11
10
9
8
7
6
5
3
2
1
0

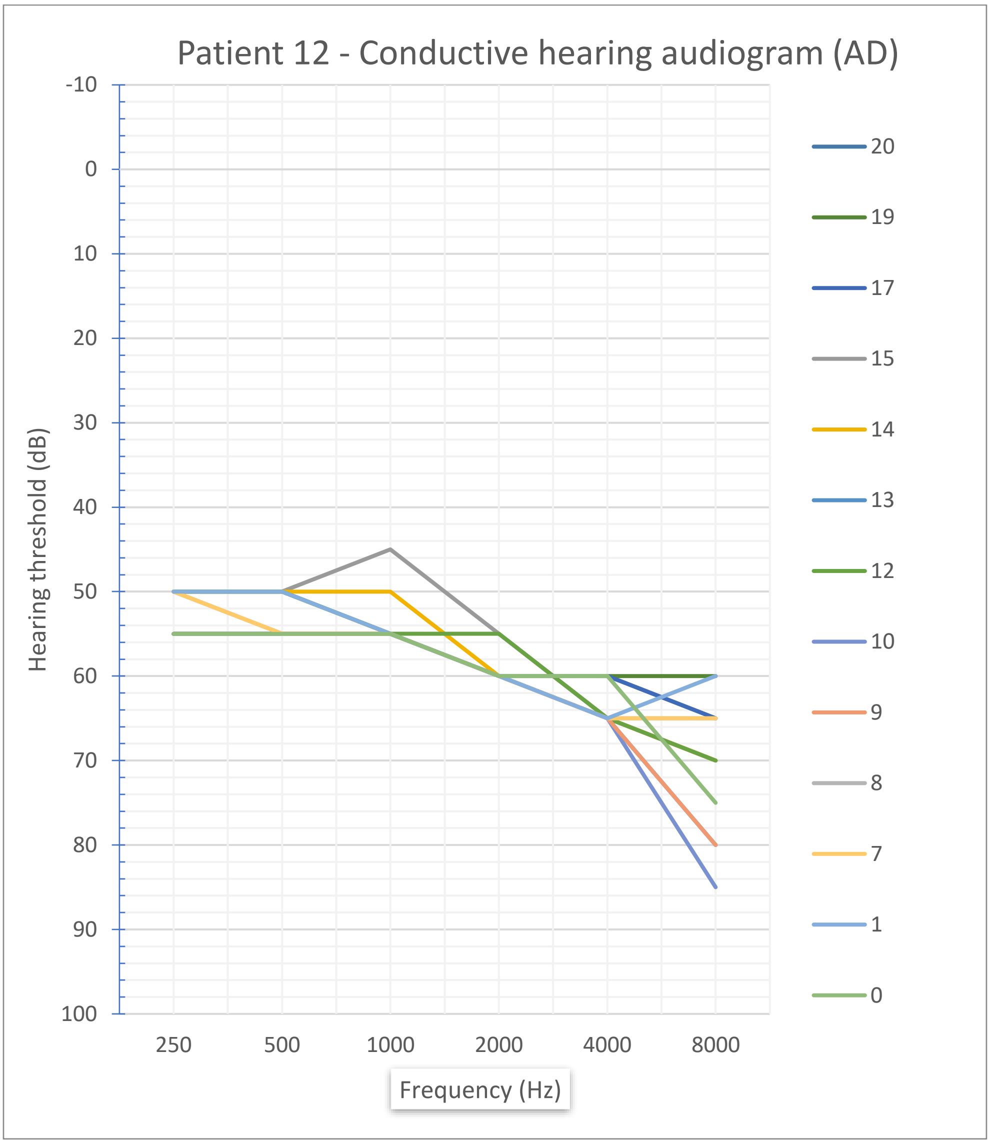

Patient 12 - Conductive hearing audiogram (AD)
Hearing threshold (dB)
-10
0
10
20
30
40
50
60
70
80
90
100
250
500
1000
2000
4000
8000
Frequency (Hz)
20
19
17
15
14
13
12
10
9
8
7
1
0

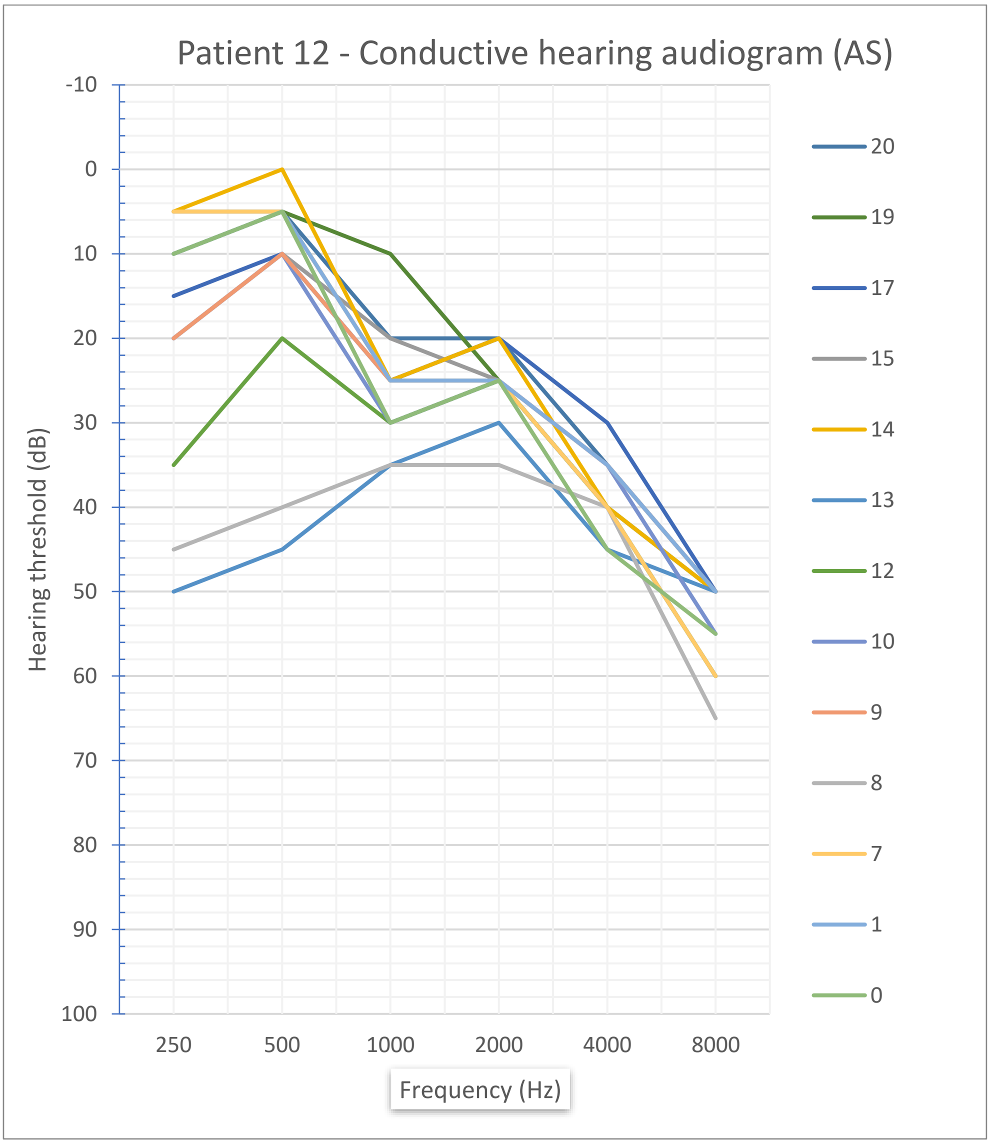
Patient 12 - Conductive hearing audiogram (AS)
Hearing threshold (dB)
-10
0
10
20
30
40
50
60
70
80
90
100
250
500
1000
2000
4000
8000
Frequency (Hz)
20
19
17
15
14
13
12
10
9
8
7
1
0

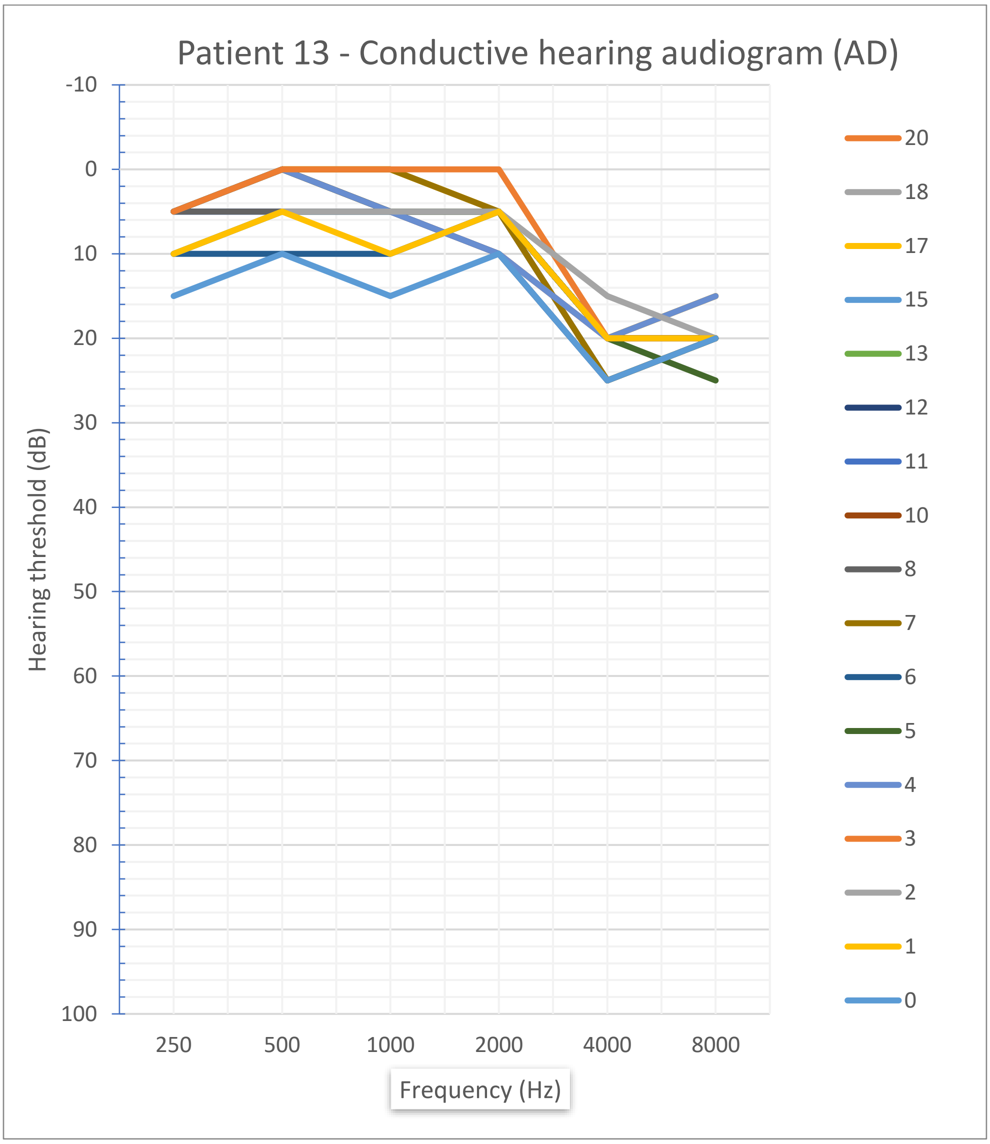

Patient 13 - Conductive hearing audiogram (AD)
Hearing threshold (dB)
-10
0
10
20
30
40
50
60
70
80
90
100
250
500
1000
2000
4000
8000
Frequency (Hz)
20
18
17
15
13
12
11
10
8
7
6
5
4
3
2
1
0

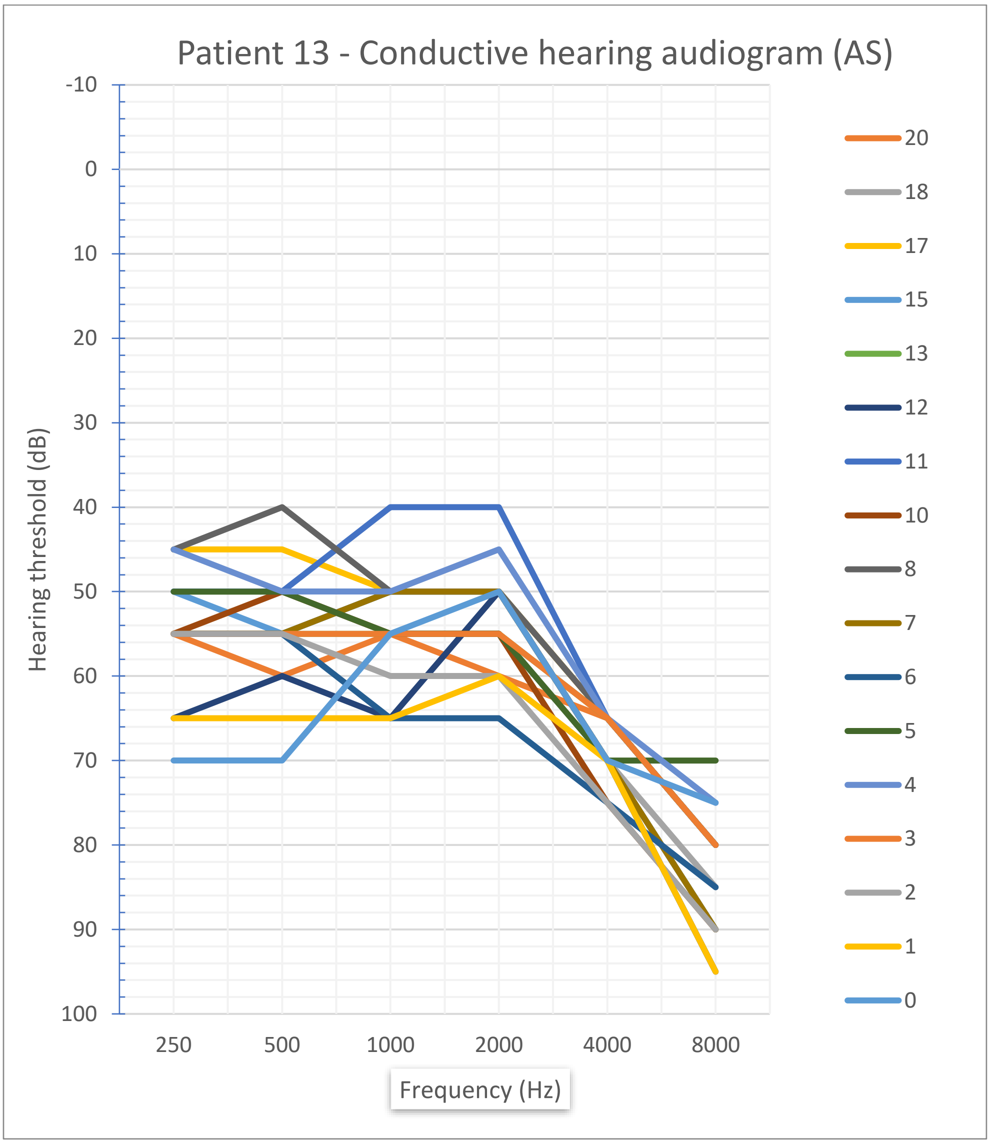
Patient 13 - Conductive hearing audiogram (AS)
-10
0
10
20
30
40
50
60
70
80
90
100
Hearing threshold (dB)
250
500
1000
2000
4000
8000
Frequency (Hz)
20
18
17
15
13
12
11
10
8
7
6
5
4
3
2
1
0

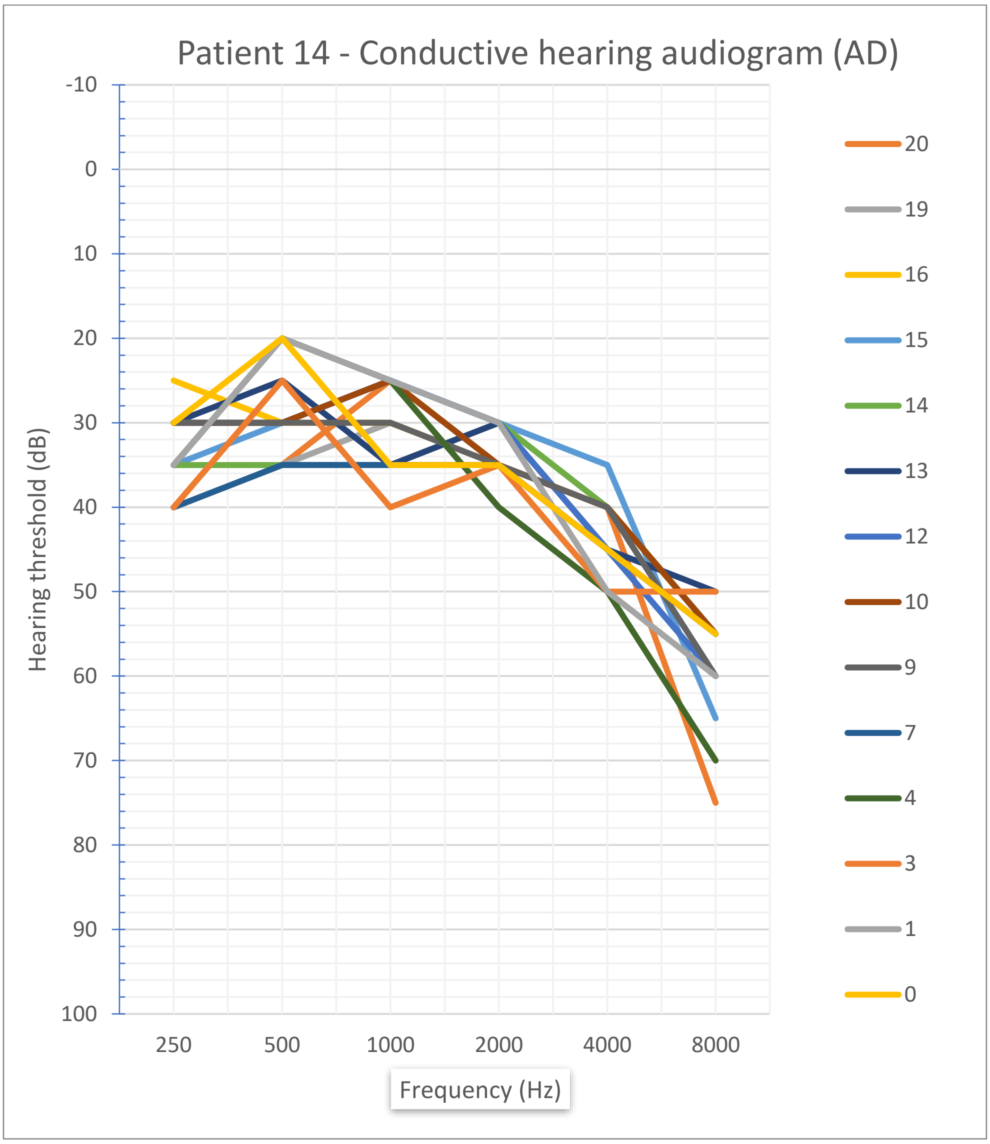

Patient 14 - Conductive hearing audiogram (AD)
Hearing threshold (dB)
-10
0
10
20
30
40
50
60
70
80
90
100
250
500
1000
2000
4000
8000
Frequency (Hz)
20
19
16
15
14
13
12
10
9
7
4
3
1
0

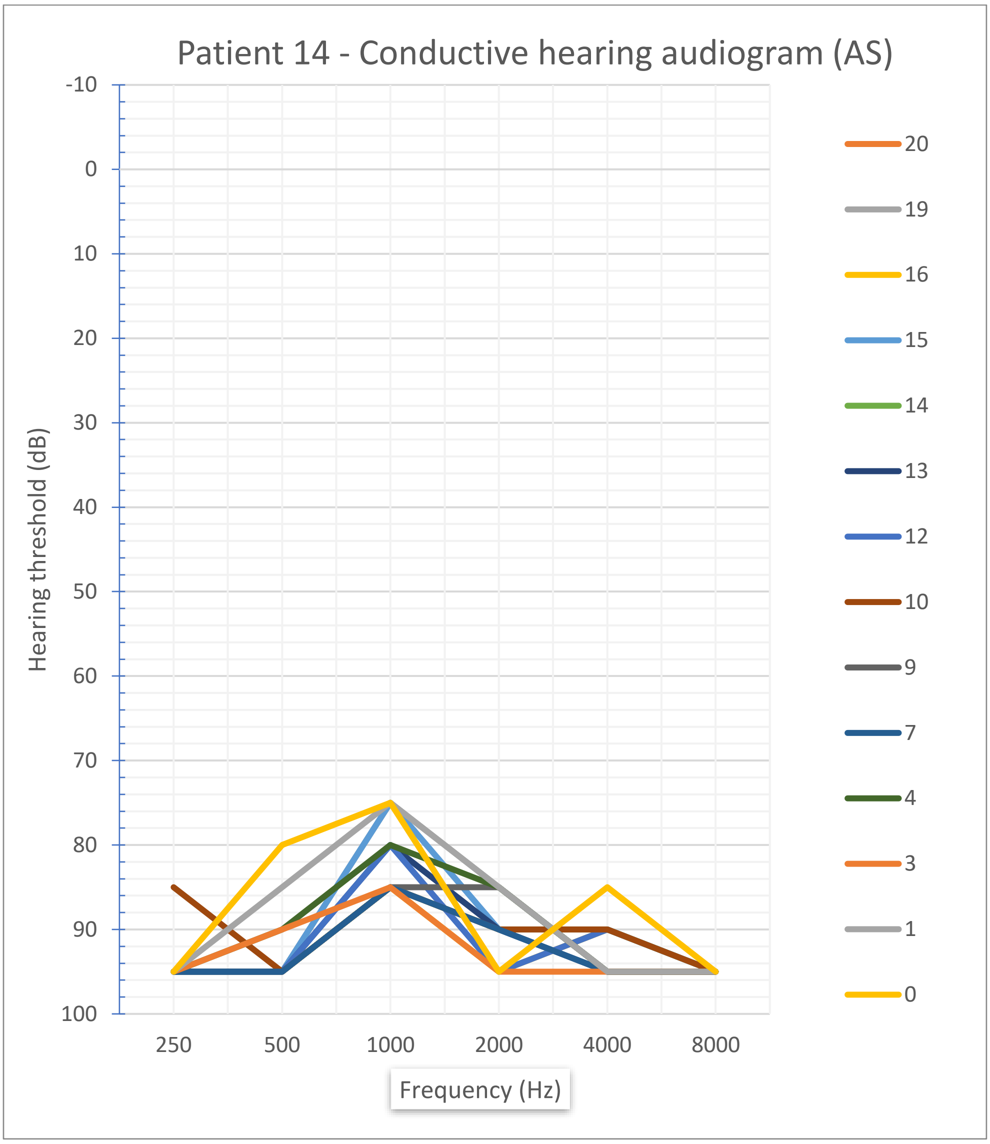

Patient 14 - Conductive hearing audiogram (AS)
Hearing threshold (dB)
-10
0
10
20
30
40
50
60
70
80
90
100
250
500
1000
2000
4000
8000
Frequency (Hz)
20
19
16
15
14
13
12
10
9
7
4
3
1
0

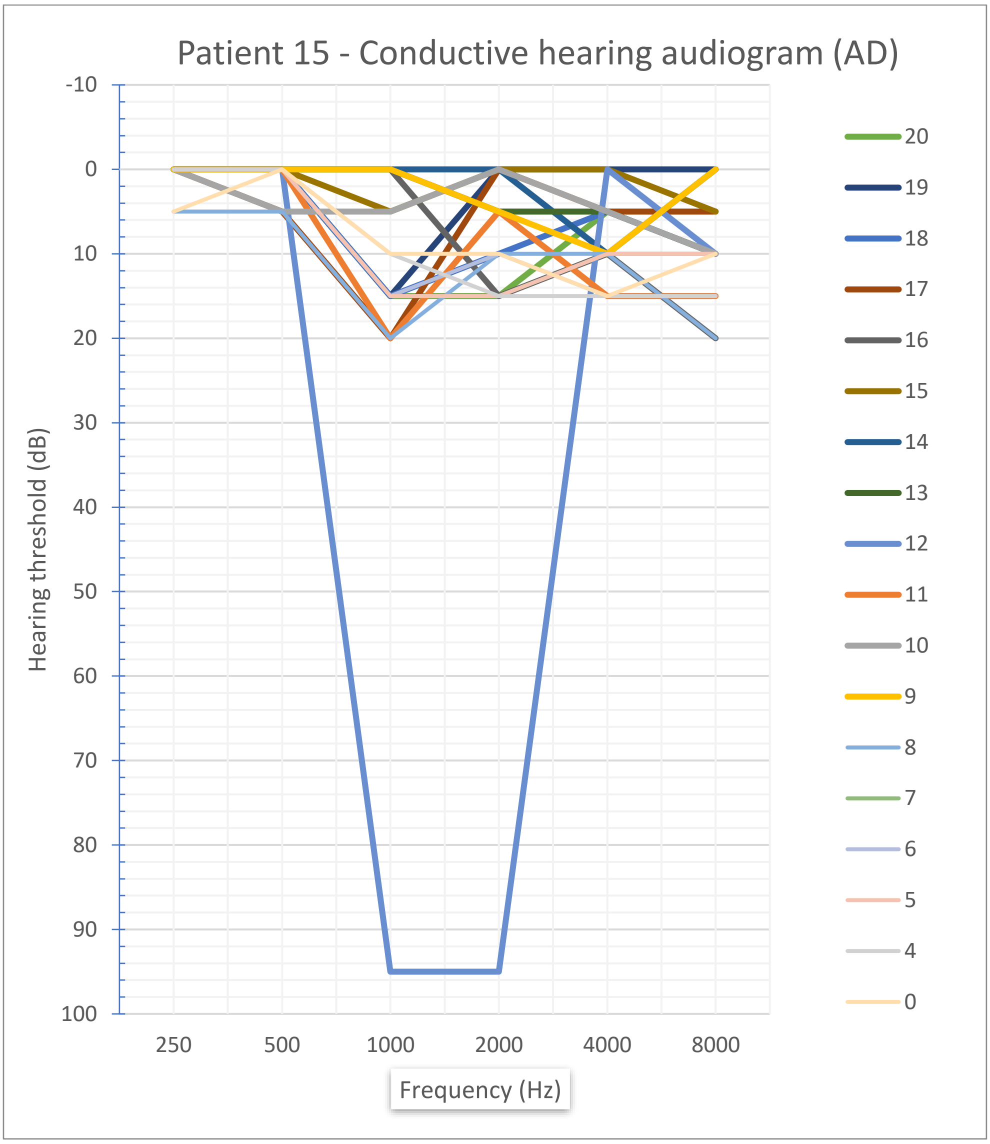

Patient 15 - Conductive hearing audiogram (AD)
-10
0
10
20
30
40
50
60
70
80
90
100
Hearing threshold (dB)
250
500
1000
2000
4000
8000
Frequency (Hz)
20
19
18
17
16
15
14
13
12
11
10
9
8
7
6
5
4
0

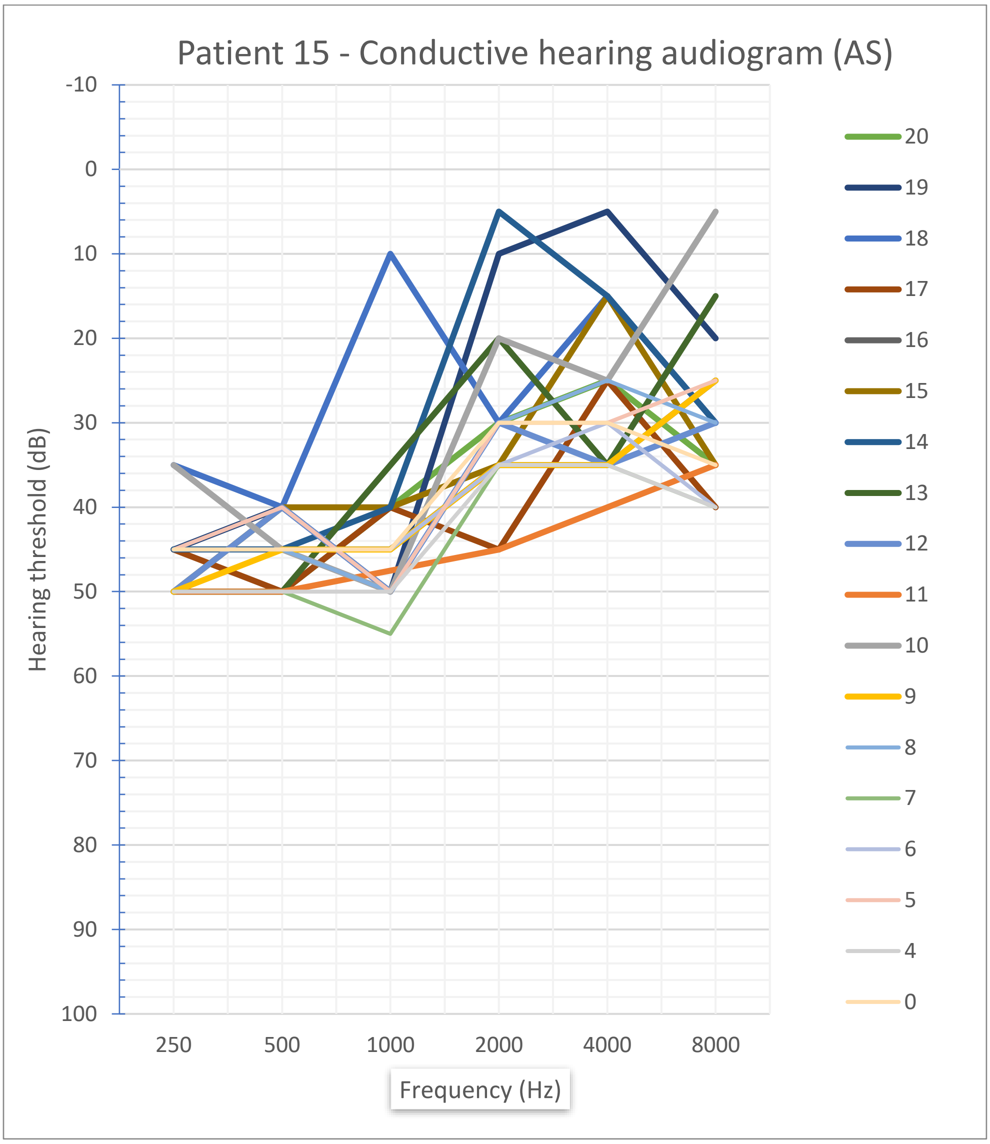
Patient 15 - Conductive hearing audiogram (AS)
-10
0
10
20
30
40
50
60
70
80
90
100
Hearing threshold (dB)
250
500
1000
2000
4000
8000
Frequency (Hz)
20
19
18
17
16
15
14
13
12
11
10
9
8
7
6
5
4
0
